# Steric‐Pressure‐Driven Topological Reconstruction for High‐Performance Zintl Phase Thermoelectrics with Machine‐Learning Validation

**DOI:** 10.1002/advs.78519

**Published:** 2026-10-26

**Authors:** Siyeon Kim, Aziz Ahmed, Juwon Lee, Jihyun Lee, Ji Hee Pi, Kyu Hyoung Lee, Kang Min Ok, Tae‐Soo You

**Affiliations:** ^1^ Department of Chemistry Chungbuk National University Cheongju Chungbuk Republic of Korea; ^2^ Department of Chemistry Sogang University Seoul Republic of Korea; ^3^ Department of Materials Science and Engineering Yonsei University Seoul Republic of Korea

**Keywords:** density functional theory, electronic structure, machine‐learning, single‐crystal X‐ray diffraction, structural transformation, thermoelectric materials, zintl phase

## Abstract

The Zintl phase A_9_M_4+_ *_x_* Pn_9_ (A = Ca, Sr, Eu, Yb; M = transition metal; Pn = pnictogen) family is a promising class of thermoelectric (TE) materials, but conventional optimization has relied on interstitial site tuning rather than structural reconstruction. In this work, we achieve a steric‐pressure‐driven topological transformation in the quinary Ca_9−_ *_x_* Sr*_x_* Zn_4+_ *_y_*_−_ *_z_* Ag*_z_* Sb_9_ system through synergistic Sr and Ag co‐doping. Single‐crystal X‐ray diffraction reveals that substituting larger Sr^2+^ for Ca^2+^ progressively drives Zn migration, transforming the parent 2‐dimensional (2D) zigzag‐shaped anionic layers into a cross‐linked 3D polyanionic framework. Density functional theory calculations indicate that Ag incorporation preserves the pseudogap near the Fermi level and reduces bond polarity, optimizing the electronic structure for hole transport. Together with the reconstructed 3D framework, these electronic effects significantly enhance carrier mobility and electrical conductivity without sacrificing a large Seebeck coefficient. As a result, the fully transformed quinary compound Ca_7.59(2)_Sr_1.41_Zn_4.37(2)_Ag_0.19_Sb_9_ achieves a maximum *ZT* of 0.73 at 873 K, doubling the performance of the parent ternary Ca_9_Zn_4.5_Sb_9_. A hybrid transfer machine‐learning approach (CrabNet and XGBoost) validates compositional trends and identifies the high‐performance region. These findings demonstrate that steric‐pressure‐induced 2D‐to‐3D framework reconstruction coupled with electronic optimization offers an effective design principle for high‐performance Zintl TE materials.

## Graphical abstract

Synergistic Sr and Ag co‐doping drives steric‐pressure‐induced reconstruction from a 2D layered structure to a cross‐linked 3D framework in the Ca_9‐_ *_x_* Sr*_x_* Zn_4+_ *_y_*_‐_ *_z_* Ag*_z_* Sb_9_ system, enhancing carrier mobility while maintaining a large Seebeck coefficient and doubling *ZT* to 0.73 at 873 K, with hybrid transfer machine learning further capturing the compositional trends and high‐performance region.

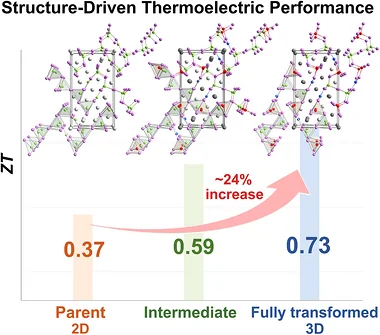

## 1 Introduction

In recent years, the design of advanced thermoelectric (TE) materials has been significantly accelerated by comprehensive strategies aimed at decoupling interdependent electrical and thermal transport properties. As highlighted in several reviews, state‐of‐the‐art material design now relies on synergistic approaches from band engineering and defect manipulation to dynamic structural phase transitions and complex framework modification to maximize the overall figure‐of‐merit *ZT* [1]. Among the various TE material classes explored for these advanced design strategies, Zintl phases have emerged as a premier candidate due to their intrinsic “phonon‐glass electron‐crystal” nature, in which a covalently bonded polyanionic framework provides electronic mobility, and the complex crystal structure effectively scatters phonons [2, 3].

Within this scope, the Zintl phase A_9_M_4+_ *_x_* Pn_9_ (A = Ca, Sr, Eu, Yb; M = transition metal; Pn = pnictogen) family offers exceptional chemical flexibility and promising TE properties [4, 5, 6, 7]. Although traditional optimization of this Zintl family has focused heavily on tuning the partially occupied interstitial sites to adjust carrier concentration [8, 9], the flexible anionic frameworks in this structure provide an untapped avenue for macroscopic material design. In particular, a critical structural insight was recently reported by Wang et al. in the Ca_9‐_ *_x_* RE*_x_* Mn_4_Sb_9_ (RE = rare earth metals) system [10], where the crystal structure was refined as the “intermediate phase” displaying a superposition of two distinct atomic arrangements as the smaller trivalent RE^3+^ substituted the divalent Ca^2+^ according to the single‐crystal X‐ray diffraction (SXRD) analysis. This observation suggests that dopant size can serve as an important tunable parameter for generating steric strain, enabling controlled manipulation of structural topology beyond conventional interstitial‐site tuning strategies [9, 10]. In addition, this study clearly indicated that intentionally applied steric pressure via targeted cation substitution could deliberately engineer the entire macroscopic crystal structure into a new topological state. Another study on the Ca_9‐_ *_x_* M*_x_* Zn_4.5_Sb_9_ (M = Sr, Eu) system also proved that Ca^2+^ substitution with larger cations, such as Sr^2+^ or Eu^2+^, expands the unit cell, reduces hole carrier concentrations, increases carrier mobility, and eventually influences TE performance via structural transformation [11, 12]. Furthermore, the *p*‐type Ag doping on the interstitial Zn site in the Ca_9_Zn_4.5‐_ *_x_* Ag*_x_* Sb_9_ system [13, 14] effectively pushes the Fermi level (*E*_F_) toward the valence band region, nearly doubling the figure‐of‐merit *ZT* value of 0.63.

Motivated by these structural clues of large‐cation expansion and the electronic benefits of Ag doping, we introduce the quinary Ca_9‐_ *_x_* Sr*_x_* Zn_4+_ *_y_*_‐_ *_z_* Ag*_z_* Sb_9_ system to engineer both the structural topology and the transport properties. By systematically replacing Ca^2+^ with the significantly larger Sr^2+^ cation, we intentionally expand the cationic sites, thereby imposing steric constraints. This targeted expansion initially drives a gradual migration of a particular Zn in the [ZnSb_4_] tetrahedron to the neighboring empty site resulting in the “intermediate phase” [10], then the accumulated steric strain forces a complete Zn migration, causing a topological reconstruction and eventually stabilizing the previously elusive “imaginary” phase with full Zn occupancy at the new atomic site [10]. Consequently, the initial 2D “zigzag‐shaped” polyanionic layered structures are transformed into fully cross‐linked, three‐dimensional (3D) complex anionic frameworks. To comprehensively map the 2D‐to‐3D structural evolution and its impact on TE performance, we conducted a series of experimental and theoretical investigations, including powder X‐ray (PXRD) and SXRD analyses, density functional theory (DFT) calculations, and a complete suite of electrical and thermal transport property measurements. Furthermore, while artificial intelligence (AI) and machine learning (ML) methods are increasingly used to analyze complex data [7, 15], the synergy between experimental practice and computational validation remains challenging. Many contemporary studies focus exclusively on using ML to predict novel, unsynthesized compounds [16, 17], while a significant opportunity exists to utilize these powerful tools to interpret, validate, and extract deeper physical insights from complex experimental results. To advance the TE research field forward and complete this synergistic loop, we expanded the experimental and theoretical investigations using our custom compositionally restricted attention‐based network (CrabNet) [18] and extreme gradient boosting (XGBoost) regressors [19] hybrid transfer ML method [20]. By coupling our experimental findings with trained ML models, we successfully demonstrate that predictive data‐driven approaches can validate the complex TE enhancements driven by this 2D‐to‐3D structural reconstruction.

## 2 Results and Discussion

### 2.1 Crystal Structure Analysis

The overall crystal structures of the two types of quinary title phases: the “intermediate phase” and “fully‐transformed phase”, should be discussed as derivatives of the ternary parent compound Ca_9_Zn_4.48(2)_Sb_9_ [6] for easier understanding. Thus, initially, the basic crystal structure of Ca_9_Zn_4.48(2)_Sb_9_ will be explained first, and then the structural differences between the two derivatives and the ternary parent phase will be elucidated later. The ternary Ca_9_Zn_4.48(2)_​Sb_9_​ crystallizes in the orthorhombic Ca_9_Mn_4_​Bi_9_​‐type phase (space group *Pbam*, Pearson code *oP*44, *Z* = 2) [6], containing five independent Ca sites, three Zn sites, and five Sb sites (Figure 1a). The overall structure features (1) a complex 2D anionic layered structure initially constructed from the assembly of tetrahedral [ZnSb_4_] building blocks and (2) the cations within as well as in between these anionic layers. The assembly of the overall anionic layered structure occurs through a precise sequence of linkage processes using the building blocks. As illustrated in Figure 1b, first, the series of adjacent [ZnSb_4_​] tetrahedra are linked via Sb3 through vertex sharing along the *c*‐axis direction to form 1D chains. Two such 1D chains are laterally connected through Sb5, then these doubled 1D chains are further linked to other 1D double chains in the inversion symmetry via Sb1 by corner‐sharing. This type of interconnection results in the formation of the quadrupled 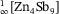 unit. Finally, cross‐linking of the neighboring quadruple units via Sb2 and Sb4 along the *a*‐axis direction binds the 1D units into a “zigzag‐shaped” 2D anionic layered structure. Interestingly, the triangular [ZnSb_3_] local coordination with the Zn partial occupations is also formed as a result of this linkage process.

**FIGURE 1:**
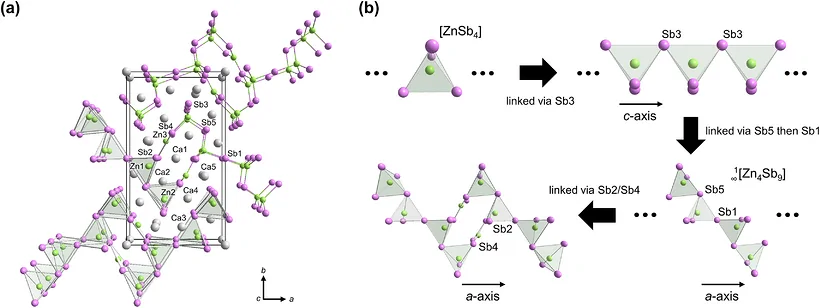
Crystal structures of (a) Ca_9_Zn_4.48(2)_Sb_9_ illustrated using ball‐and‐stick and polyhedral representations viewed along the *c*‐axis direction. (b) Schematic illustration of the hierarchical assembly of the 2D zigzag‐shaped anionic layered structure from the [ZnSb_4_] building blocks. Color codes: Ca, gray; Zn, lime green; Sb, pink.

On the other hand, in the title quinary Ca_9‐_ *_x_* Sr*_x_* Zn_4+_ *_y_*_‐_ *_z_* Ag*_z_* Sb_9_ (0.55(3) ≤ *x* ≤ 1.43(2); 0.50(3) ≤ *y* ≤ 0.56(2); 0.10(2) ≤ *z* ≤ 0.29(2)) system, the gradual substitution of Sr^2+^ for Ca^2+^ drives a sharp structural transformation from the above mentioned parental Ca_9_Mn_4_Bi_9_‐type phase [5] to the partially transformed intermediate Ca_9_Mn_4_Bi_9_‐type phase, [10] and finally to the fully transformed novel “Ca_7.6_Sr_1.4_Zn_4.4_Ag_0.2_Sb_9_‐type” phase as shown in Figure 2a,b. The detailed refinement results of SXRD data for the five title compounds are provided in Tables 1, Tables S1 and S2.

**FIGURE 2:**
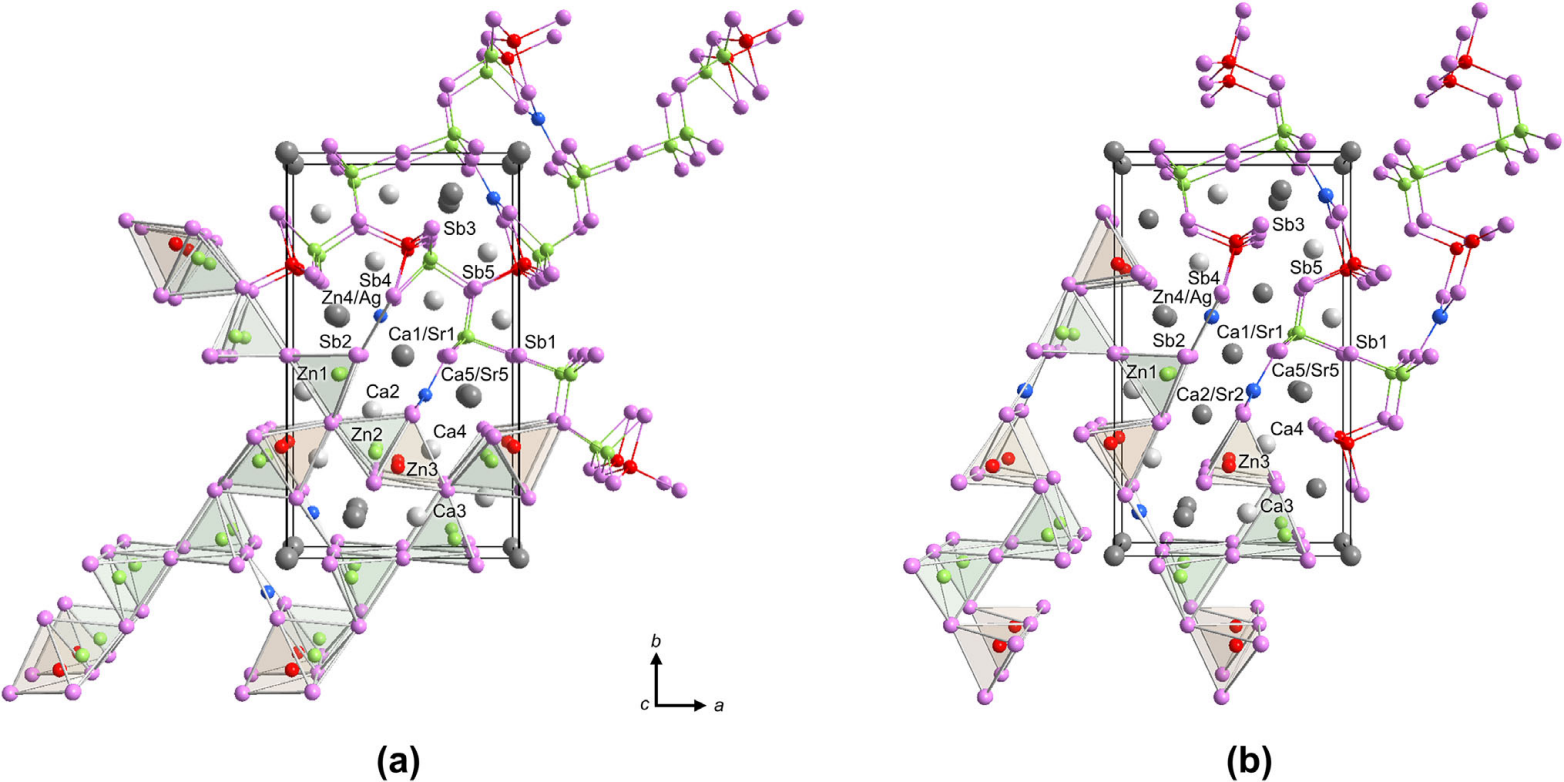
Crystal structures of (a) Ca_8.11(3)_Sr_0.89_Zn_4.33(2)_Ag_0.21_Sb_9_ (the intermediate Ca_9_Mn_4_Bi_9_‐type) and (b) Ca_7.59(2)_Sr_1.41_Zn_4.37(2)_Ag_0.19_Sb_9_ (the novel Ca_7.6_Sr_1.4_Zn_4.4_Ag_0.2_Sb_9_‐type) illustrated using polyhedral representations. The isometric tetrahedral [ZnSb_4_] building block appears as a polyhedron highlighted in light lime green and light red in both title phases. Zn3 and Zn4/Ag in (b) Ca_7.59(2)_Sr_1.41_Zn_4.37(2)_Ag_0.19_Sb_9_ are renamed as Zn2 and Zn3/Ag in the main text for consistency. Atomic labels are provided, and each unit cell is outlined in black. Color codes: Ca, gray; Ca/Sr mixed‐site, black; Zn (original), lime green; Zn (migrated), red; Zn/Ag mixed‐site, blue; and Sb, pink.

**TABLE 1:** SXRD data and structure refinement results for the Ca_9‐_ *_x_* Sr*_x_* Zn_4+_ *_y_*_‐_ *_z_* Ag*_z_* Sb_9_ (0.55(3) ≤ *x* ≤ 1.43(2); 0.50(3) ≤ *y* ≤ 0.56(2); 0.10(2) ≤ *z* ≤ 0.29(2)) system.

Empirical formula	Ca_8.45(3)_Sr_0.55_Zn_4.28(3)_Ag_0.22_Sb_9_	Ca_8.11(3)_Sr_0.89_Zn_4.33(2)_Ag_0.21_Sb_9_	Ca_7.97(3)_Sr_1.03_Zn_4.42(2)_Ag_0.10_Sb_9_	Ca_7.59(2)_Sr_1.41_Zn_4.37(2)_Ag_0.19_Sb_9_	Ca_7.57(2)_Sr_1.43_Zn_4.27(2)_Ag_0.29_Sb_9_
Crystal system	Orthorhombic				
Space group	*Pbam* (No. 55)				
Unit cell dimensions (Å)	*a* = 12.5564(15)	*a* = 12.7519(4)	*a* = 12.7666(3)	*a* = 12.7956(4)	*a* = 12.8004(11)
	*b* = 21.951(3)	*b* = 21.8014(6)	*b* = 21.8095(5)	*b* = 21.8441(6)	*b* = 21.8593(18)
	*c* = 4.5626(5)	*c* = 4.5545(2)	*c* = 4.5562(2)	*c* = 4.5605(2)	*c* = 4.5617(4)
Volume (Å^3^)	1257.6(2)	1266.2(2)	1268.6(1)	1274.7(1)	1276.4(2)
*d*_calcd_ (g/cm^3^)	4.717	4.731	4.726	4.766	4.774
Data/restraints/parameters	1621/1/83	1463/1/85	1465/1/84	1525/1/82	1262/1/83
*R* ^a^ indices (*I* > 2*σ*(*I*))	*R*_1_ = 0.0262	*R*_1_ = 0.0218	*R*_1_ = 0.0207	*R*_1_ = 0.0205	*R*_1_ = 0.0188
	*wR*_2_ = 0.0583	*wR*_2_ = 0.0477	*wR*_2_ = 0.0453	*wR*_2_ = 0.0449	*wR*_2_ = 0.0398
*R* ^a^ indices (all data)	*R*_1_ = 0.0270	*R*_1_ = 0.0258	*R*_1_ = 0.0221	*R*_1_ = 0.0238	*R*_1_ = 0.0238
	*wR*_2_ = 0.0585	*wR*_2_ = 0.0490	*wR*_2_ = 0.0458	*wR*_2_ = 0.0459	*wR*_2_ = 0.0412
Goodness‐of‐fit on *F* ^2^	1.367	1.109	1.246	1.110	1.113
Largest differences of peak/hole (e/Å^3^)	2.197/‐1.537	2.067/‐1.894	2.091/‐1.962	2.378/‐1.791	1.824/‐1.568

^a^ *R*_1_ = Σ**||** *F*_o_ **|**—**|** *F*_c_ **||**/Σ**|** *F*_o_ **|**; *wR*_2_ = (Σ(*w*(*F*_o_^2^ – *F*_c_^2^)/Σ(*w*(*F*_o_^2^)^2^))^1/2^, where *w* = 1/(*σ* ^2^ *F*_o_^2^+(A – *P*)^2^+B – *P*), in which *P* = (*F*_o_^2^ +2*F*_c_^2^)/3; A and B—weight coefficients.

The driving force for this type of structural transformation is the localized steric strain induced by the addition of a relatively larger Sr^2+^ cation (*r*(Sr^2+^) = 1.18 Å, *r*(Ca^2+^) = 1.00 Å) [21] to the Ca^2+^ sites in the title system. To minimize the initial steric strain, the incoming Sr^2+^ initially prefers to occupy the two most spacious cationic sites, Ca1 (46.99 Å^3^) and Ca5 (47.39 Å^3^), and avoid the relatively smaller Ca2 (45.01 Å^3^), Ca3 (45.02 Å^3^), and Ca4 (45.11 Å^3^) (See Figure S1). As Sr concentration increases, Sr cascades into the relatively smaller Ca2 site as provided in Table S3.

The following two‐stage transformation pathway can elucidate the observed site preference of these cations and the resultant energetic driving force for Zn migration. First, as shown in Figure 3a, in the relatively lower‐substitution regime (0.55(3) ≤ *x* ≤ 1.03(3)), the addition of a small amount of Sr initially substitutes the more spacious Ca1 and Ca5 sites with occupancies of 12% and 22% in Ca_8.45(3)_Sr_0.55_Zn_4.28(3)_Ag_0.22_Sb_9_ (See Table S1). This type of partial Sr substitution expands the volume of the Ca1(Ca1/Sr1 mixed‐site) site from 46.99 Å^3^ to 48.19 Å^3^ (See Table S3). This substantial volumetric expansion acts as a localized steric pressure, applying a repulsive interaction toward Ca2 that lengthens the adjacent Ca1–Ca2 interatomic distance from 4.14(2) Å to 4.20(1) Å in Ca_9_Zn_4.48(2)_Sb_9_ and Ca_8.45(3)_Sr_0.55_Zn_4.28(3)_Ag_0.22_Sb_9_, respectively. Then, as a result of this Ca2 shift toward the adjacent Zn2 located at the center of the [ZnSb_4_] tetrahedron, the unprecedentedly short Ca‐Zn interatomic distance of 3.17(1) Å is observed [22, 23]. To escape the severe energetic penalty caused by this spatial crowding, the central Zn2 eventually chooses partial migration to the neighboring empty Zn3 site, which is the center of a newly formed tetrahedron. This type of Zn migration results in split sites with partial occupancies of Zn2:Zn3 = 92%: 8% in the intermediate phase Ca_8.45(3)_Sr_0.55_Zn_4.28(3)_Ag_0.22_Sb_9_ as illustrated in Figure 3b. Interestingly, a discrete structural shift occurs as the Sr content increases to *x* = 0.89 in Ca_8.11(3)_Sr_0.89_Zn_4.33(3)_Ag_0.21_Sb_9_, where the Zn2:Zn3 occupancies dramatically change to 9%:91%. This rapid transition from a predominantly 2D layered network to a *ca*. 91% cross‐linked 3D framework is accompanied by a sharp contraction in the Ca2‐Sb3 distance from 3.632(2) Å to 3.344(2) Å as provided in Table S2 and Figure S2, confirming that most of the topological reconstruction is completed within this range of composition. Furthermore, as the Sr content continuously increases in this phase, the resultant partial occupancies between Zn2 and Zn3 eventually become 8% and 92%, respectively, as observed in Ca_7.97(3)_Sr_1.03_Zn_4.42(2)_Ag_0.10_Sb_9_. Moreover, this type of partial migration of Zn2 also gradually rearranges the local connectivity of the [ZnSb_4_] tetrahedron, modifying the original corner‐sharing configuration of the 1D chains into newly formed Zn3–Sb5 linkages along the migration pathway (See Figure 2b). In particular, the observed intermediate phase, where partially occupied Zn2 and Zn3 sites coexist, mirrors intermediate configurations reported earlier in the Ca_9−_ *_x_​*RE*_x_* Mn_4_Sb_9_​ (RE = La‐Nd, Sm) system [10].

**FIGURE 3:**
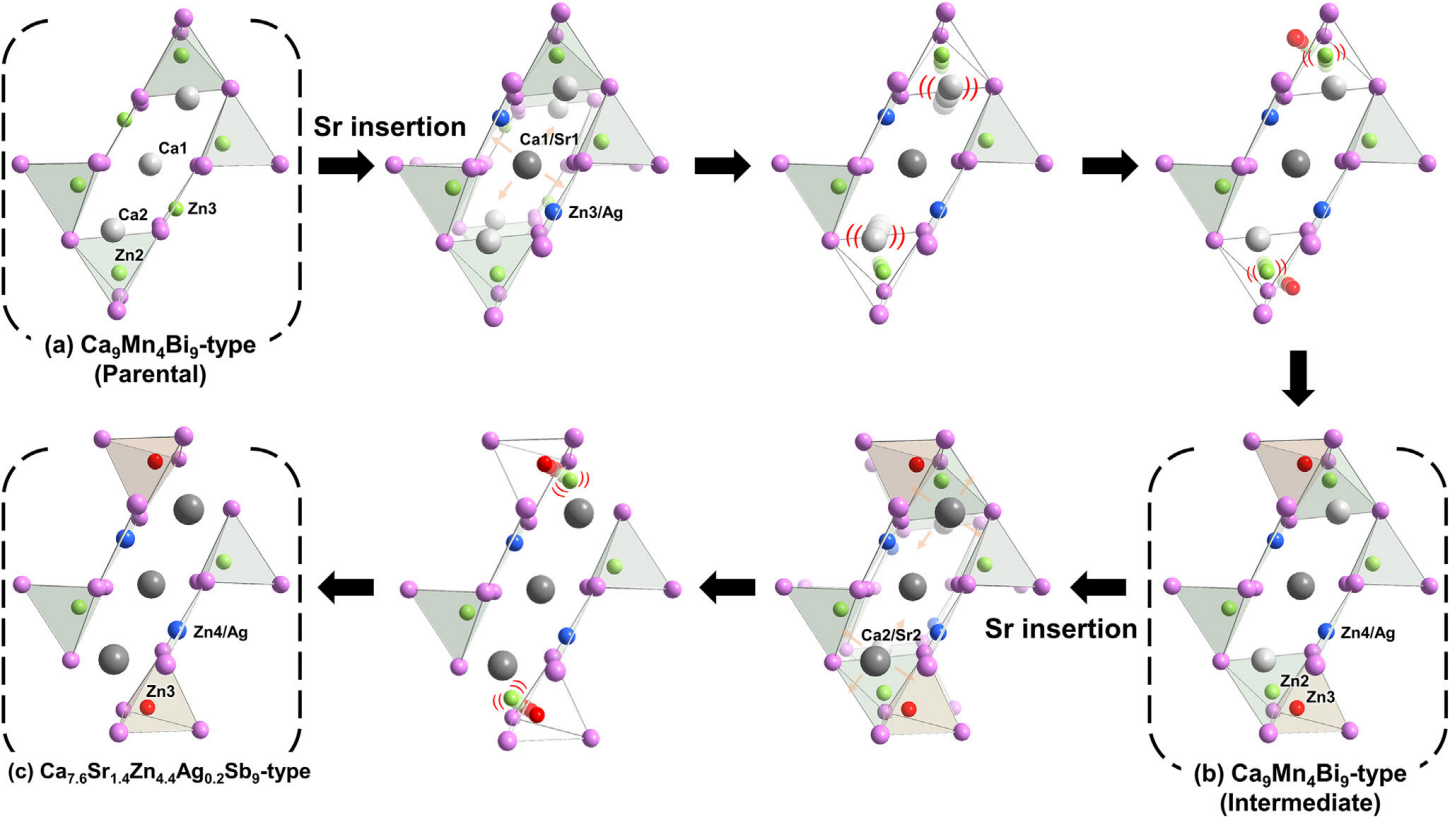
Schematic illustration showing the structural transformation process caused by Sr insertion in the title in the Ca_9‐_ *_x_* Sr*_x_* Zn_4+_ *_y_*_‐_ *_z_* Ag*_z_* Sb_9_ system from (a) the parental Ca_9_Mn_4_Bi_9_‐type to (b) the intermediate Ca_9_Mn_4_Bi_9_‐type, and finally (c) the novel Ca_7.6_Sr_1.4_Zn_4.4_Ag_0.2_Sb_9_‐type structure. Zn3 and Zn4/Ag in the novel Ca_7.6_Sr_1.4_Zn_4.4_Ag_0.2_Sb_9_‐type phase are renamed as Zn2 and Zn3/Ag in the main text for consistency. Selected atomic labels are also provided.

Second, the high‐substitution regime (*x* ≥ 1.41) represents the completion threshold, where Sr begins to directly occupy the Ca2 site as well, triggering an additional expansion of the Ca2 volume from 45.01 to 46.25 Å^3^ (Table S3). This kind of polyhedral expansion maximizes the energetic penalty on the adjacent Zn2 atom. Therefore, to relieve the thermodynamically unfavorable steric pressure and minimize the total energy of the lattice, the given intermediate structure eventually allows complete Zn migration from the constrained Zn2 site to the less stressed Zn3 site as illustrated in Figure 3c. This kind of atomic relocation perfectly coincides with the macroscopic transformation to fully connected 3D anionic frameworks, quantitatively confirming steric strain as the fundamental energetic trigger for topological reconstruction. As a result, the original Zn2 site becomes completely vacant, achieving a 100% fully connected 3D framework without any remaining 2D characteristics, and eventually marks the first demonstration of Sr‐induced complete reconstruction of the anionic framework. We call this novel crystal structure the “Ca_7.6_Sr_1.4_Zn_4.4_Ag_0.2_Sb_9_‐type” phase. For crystallographic consistency, the newly formed Zn3 and triangular Zn4 sites are renamed Zn2 and Zn3 sites in the novel phase, respectively, at the final stage of structure refinement. Topologically, this Zn atom migration transforms the original zigzag‐shaped 2D anionic layered structure with no Zn‐Sb linkages along the *b*‐axis direction into a fully connected 3D polyanionic framework featuring a cross‐linked Sb4‐Zn2‐Sb5‐Zn1 connection along the *b*‐axis direction, which eventually influences the electrical conductivity of the novel phase. We will further discuss carrier mobility (*µ*) in the Thermoelectric Properties Measurements section.

### 2.2 Electronic Structure Analysis

#### 2.2.1 Density of States and Chemical Bonding

To understand the electronic structure changes induced by Sr and Ag co‐doping in the title Ca_9‐_ *_x_* Sr*_x_* Zn_4+_ *_y_*_‐_ *_z_* Ag*_z_* Sb_9_ system, a series of DFT calculations was performed on three hypothetical models using the tight‐binding linear muffin‐tin orbital (TB‐LMTO) method with atomic sphere approximation (ASA) [24, 25, 26]. These structural models were designed to represent the observed three distinct title phases: (1) the ternary parent phase Ca_9_Zn_4.5_Sb_9_, (2) the quinary intermediate phase Ca_8_SrZn_4.25_Ag_0.25_Sb_9_, and (3) the fully transformed quinary novel phase Ca_7.5_Sr_1.5_Zn_4.25_Ag_0.25_Sb_9_, and their electronic structures were carefully calculated and compared. To properly assign the experimentally observed mixed occupancies of Zn and Ag in these quinary models, the SXRD refined space group *Pbam* (No. 55) was lowered to its subgroup *Pm* (No. 6). Further details about these three structural models are provided in Section S1 and Table S4.

Although these hypothetical models provide crucial insights into the fundamental orbital interactions, it is important to consider the inherent limitations of this DFT calculation approach. Experimentally synthesized solid‐solution products naturally possess configurational disorder, local lattice strain fluctuations, and non‐ideal defect concentrations that cannot be perfectly applied to the fully ordered stoichiometric hypothetical models with lowered symmetry in DFT approaches. Thus, these calculations represent only an idealized approximation of the experimentally obtained compounds. However, it should be noted that the dynamical and thermodynamic stability of these reconstructed frameworks is unequivocally confirmed by our successful experimental synthesis and stable high‐temperature TE measurements up to *ca*. 870 K, which rule out the existence of structural instabilities or imaginary phonon modes in the experimentally obtained real products. Furthermore, because the models preserve the average coordination geometries and substitute elements with the correct valence and orbital characteristics, they reliably capture the overall trends in orbital mixing, pseudogap formation, and bond polarity changes. Since the overall features of the density of states (DOS) and crystal orbital Hamilton population (COHP) curves of the two quinary models resemble each other, as given in Figure S3, we mainly focus on the calculation results of the undoped parent phase Ca_9_Zn_4.5_Sb_9_ and the Sr‐ and Ag‐co‐doped novel phase Ca_7.5_Sr_1.5_Zn_4.25_Ag_0.25_Sb_9_ as shown in Figure 4.

**FIGURE 4:**
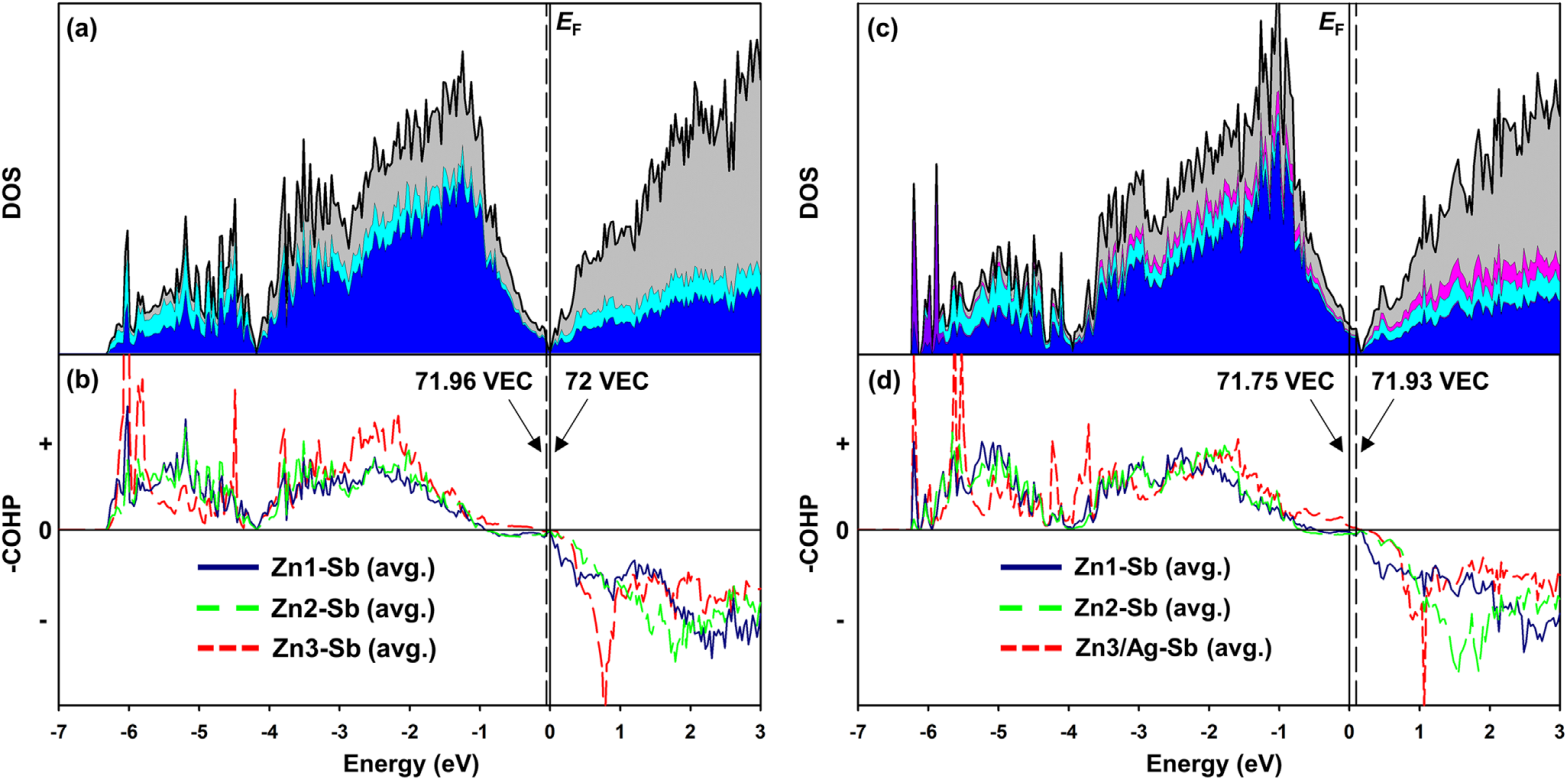
TDOS, PDOS, and COHP of Ca_9_Zn_4.5_Sb_9_ (a,b) and Ca_7.5_Sr_1.5_Zn_4.25_Ag_0.25_Sb_9_ (c,d). In the COHP diagram, the “+” region indicates bonding interactions, while the “‐” region represents antibonding interactions. Color codes in the DOS curve are as follows: TDOS, black outline; Ca PDOS, gray region; Sr PDOS, pink region; Zn PDOS, cyan region; Ag PDOS, purple region; and Sb PDOS, blue region. *E*_F_ (vertical line) is set as the energy reference at 0 eV, and the corresponding VEC is also displayed. The VECs of 71.96 and 71.93, corresponding to the refined compositions of Ca_9_Zn_4.48(2)_Sb_9_ and Ca_7.59(2)_Sr_1.41_Zn_4.37(2)_Ag_0.19_Sb_9_, respectively, are also displayed.

First, the calculated total and partial DOS (TDOS and PDOS) of the ternary parent phase Ca_9_Zn_4.5_Sb_9_ and the fully transformed 3D novel phase Ca_7.5_Sr_1.5_Zn_4.25_Ag_0.25_Sb_9_ show strong overall orbital mixing among the constituent elements, as displayed in Figure 4a,c. Both TDOS curves also exhibit a pronounced pseudogap near the Fermi level (*E*_F_​), which is characteristic of heavily doped semiconducting or semi‐metallic compounds [27, 28]. In particualr, the DOS curves in valence band region between *ca*. −6.5 and 0 eV in these two models can briefly be divided into two sectors: (1) the lower sector between *ca*. −6.5 and ‐4.5 eV is mostly contributed by the *p*‐states of Sb2, Sb3, and Sb5 forming the relatively shorter Zn‐Sb distances (2.648(2) ∼ 2.842(2) Å) in the tetrahedral [ZnSb_4_] moiety with small contributions from Zn, whereas (2) the upper sector between *ca*. −4.5 and 0 eV contains the *p*‐states contributions from all Zn and Sb with some additional contributions from the mixtures of cationic elements. Second, COHP curve [29] analysis further reveals the characteristics of several chemical bonds observed in the anionic frameworks (Figure 4b,d). In both structural models, the averaged Zn1‐Sb and Zn2‐Sb COHP curves representing chemical bonds in the tetrahedral [ZnSb_4_] moiety display very similar patterns, which are optimized at *E*_F_ and show a strong antibonding character beyond *E*_F_. However, the averaged Zn3‐Sb and Zn3/Ag‐Sb COHP curves in the Ca_9_Zn_4.5_Sb_9_ and Ca_7.5_Sr_1.5_Zn_4.25_Ag_0.25_Sb_9_ models, respectively, represent interatomic interactions in the triangular [Zn3(Zn3/Ag)Sb_3_] moiety, and show slightly different behavior. As the Ag dopant is introduced for Zn in the quinary model, the Zn3/Ag‐Sb bond strength becomes relatively weaker compared to that of the ternary model due to the elongated Zn‐Sb distances (2.561(2)∼2.652(2) Å) as provided in Table S2. To quantitatively understand this difference, the corresponding integrated COHP (ICOHP) values were evaluated and compared: −2.350 eV for Zn3–Sb in Ca_9_Zn_4.5_Sb_9_ and −2.182 eV for Zn3/Ag–Sb in Ca_7.5_Sr_1.5_Zn_4.25_Ag_0.25_Sb_9_​. The observed ICOHP value decreases from 2.350 to 2.182 eV, supporting the relative weakening of the corresponding bonding interactions in the co‐doped model. As a result, room for additional electrons is generated at the top of the valence band region [30]. Thus, it is clear that the *p*‐type Ag dopant successfully generates some empty states for additional electrons just below *E*_F_.

To further understand the discrete effects of each dopant, two hypothetical models having the ‘Sr‐only’ doping (Ca_8_SrZn_4.5_Sb_9_) and the ‘Ag‐only’ doping (Ca_9_Zn_4.25_Ag_0.25_Sb_9_) were carefully studied, and the resultant DOS and COHP curves are displayed in Figure S4. The Sr‐only model exhibits slightly increased antibonding character near and above *E*_F_​ in the COHP curves, implying that the Sr‐driven reconstruction may introduce local electronic instability. On the other hand, the Ag‐only model demonstrates a clear shift of *E*_F_ into the valence band region along with somewhat extended non‐bonding character in the Zn/Ag‐Sb bond. Thus, although Sr causes the steric‐strain‐driven structural transformation, Ag acts as a crucial electronic stabilizer by providing necessary hole doping. In addition, Ag doping generates some empty states just below *E*_F_​, which perfectly reconciles with our experimental Hall effect measurements discussed in Section 2.3.

#### 2.2.2 Electron Localization Function (ELF) Analysis

A series of ELF calculations were also performed for the parental phase Ca_9_Zn_4.5_Sb_9_ and the novel phase Ca_7.5_Sr_1.5_Zn_4.25_Ag_0.25_Sb_9_ models to understand the influence of the Ag doping for Zn at the center of triangular coordinates in terms of the bond polarity and the resultant *µ* within the anionic frameworks. As shown in Figure 5, in the ELF isosurfaces of both models, the two lone pairs on each of the bridging Sb3 and Sb4 are represented by C‐shaped isosurfaces as previously observed in the Ca_3‐_ *_x_* Yb*_x_* AlSb_3_ and Ca_9_Cd_3+_ *_x_*_‐_ *_y_* M*_x_*_+_ *_y_* Sb_9_ (M = Cu, Zn) systems [31, 32]. However, as the central Zn in the triangular [ZnSb_3_] moiety (Figure 5a) was substituted by Ag as in the quinary model, the C‐shaped lone pairs on Sb4 were strongly withdrawn toward the replacing Ag as shown in Figure 5b. This type of distortion of lone pairs should be attributed to the larger electronegativity of Ag than Zn, resulting in a reduction of the electronegativity difference between two anionic elements on the anionic frameworks (Zn = 1.65, Ag = 1.93, and Sb = 2.05 in the Pauling scale) [33]. The Pauling electronegativity differences are *Δ*Zn−Sb = 0.40 and *Δ*Ag−Sb = 0.12. Thus, Ag doping can decrease the electronegativity difference between two anionic elements by 0.28. This metric provides a numerical basis for the proposed decrease in local bond polarity, while the ELF analysis provides complementary evidence in paired electron densities. Therefore, the observed redistribution of lone pairs represents a decrease in overall bond polarity within the anionic frameworks [6, 7, 34], which eventually serves as a primary mechanism for the dramatic enhancement of hole *µ* in the quinary title compounds as reported in Table 2. Further details on hole carrier concentration and mobility will be provided in the subsequent Thermoelectric Properties Measurement section.

**FIGURE 5:**
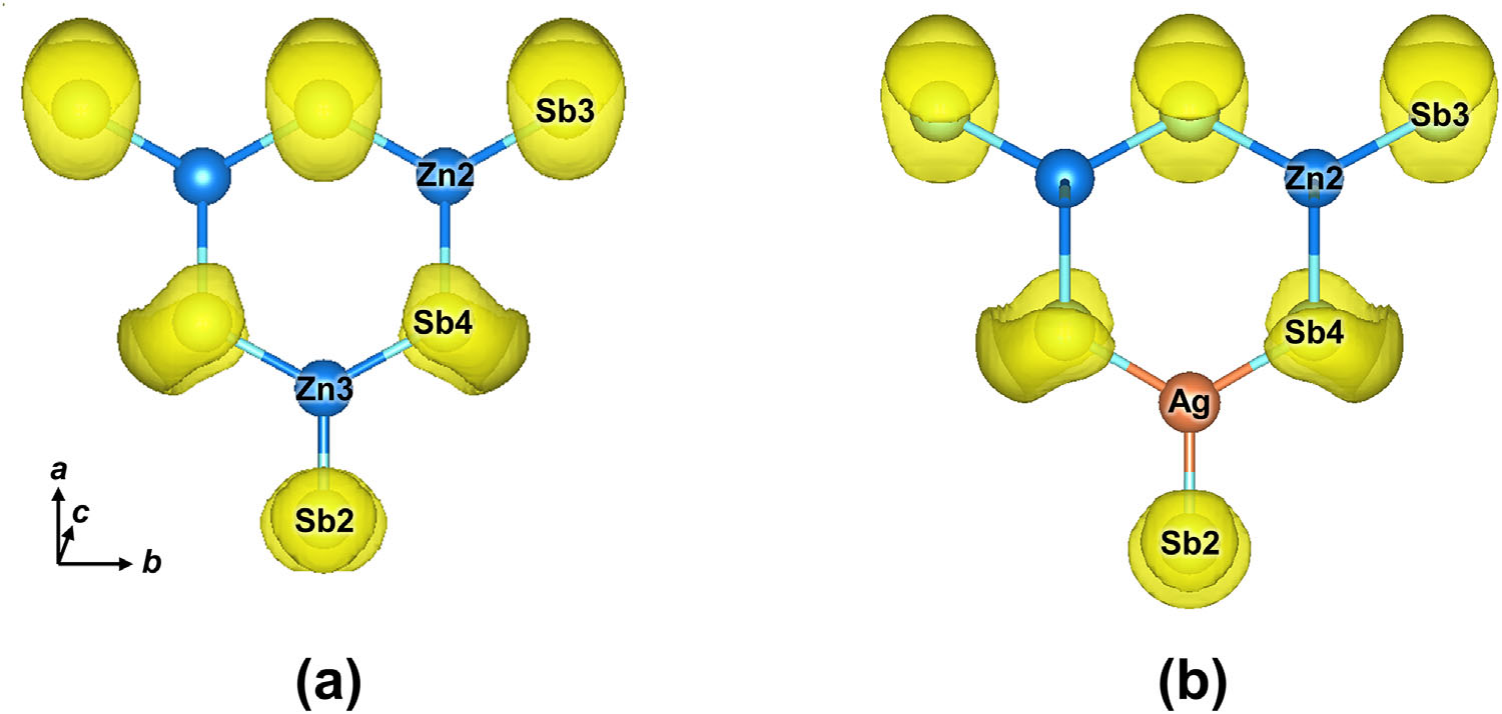
The 3D ELF isosurfaces for the partial anionic frameworks in (a) Ca_9_Zn_4.5_Sb_9_ and (b) Ca_7.5_Sr_1.5_Zn_4.25_Ag_0.25_Sb_9_. The isosurfaces correspond to an ELF value of 0.64.

**TABLE 2:** Carrier concentration, mobility, bandgap, and effective mass of selected compounds in the Ca_9‐_ *_x_* Sr*_x_* Zn_4+_ *_y_*_‐_ *_z_* Ag*_z_* Sb_9_ (0 ≤ *x* ≤ 1.43(2); 0.50 ≤ *y* ≤ 0.56(2); 0 ≤ *z* ≤ 0.29(2)) system.

Compound	Carrier concentration *n* (× 10^19^ carriers/cm^3^)	Mobility *µ* (cm^2^/Vs)	Bandgap (eV)	Effective mass (*m* ^*^/*m*_e_)
Ca_9_Zn_4.5_Sb_9_ [8]	6.07	48.97	0.25	0.46
Ca_7.97(3)_Sr_1.03_Zn_4.42(2)_Ag_0.10_Sb_9_	4.88	74.62	0.25	0.56
Ca_7.59(2)_Sr_1.41_Zn_4.37(2)_Ag_0.19_Sb_9_	4.66	98.93	0.26	0.63
Ca_7.57(2)_Sr_1.43_Zn_4.27(2)_Ag_0.29_Sb_9_	5.60	87.24	0.25	0.76

Finally, although ELF and COHP analyses provide a qualitative visualization of chemical bonding, these localized electronic structure modifications directly affect the quantitative macroscopic TE transport properties discussed in Section 2.3. The decrease in bond polarity observed in the ELF isosurfaces suppresses carrier scattering, directly contributing to the sharp *µ* increase observed in the fully transformed 3D phases. Furthermore, the topological reconstruction can alter the global band structure, which is experimentally quantified by a substantial increase in effective mass (*m* ^∗^) from 0.46 *m*_e_ to 0.76 *m*_e_​ evaluated using the Mott relationship. This structurally induced band structure modification, coupled with the hole‐doping via the Ag addition as confirmed by the DFT calculation for the Ag‐only model, serves as the fundamental driving force for the enhanced Seebeck coefficient (*S*) in the quinary compounds.

### 2.3 Thermoelectric Properties Measurement

To evaluate the macroscopic impact of the Sr‐ and Ag‐co‐doping strategy, the TE transport properties of the five title compounds in the Ca_9‐_ *_x_* Sr*_x_* Zn_4+_ *_y_*_‐_ *_z_* Ag*_z_* Sb_9_ (0.55(3) ≤ *x* ≤ 1.43(2); 0.50(3) ≤ *y* ≤ 0.56(2); 0.10(2) ≤ *z* ≤ 0.29(2)) system were systematically investigated. By measuring the electrical conductivity *σ*, the Seebeck coefficient *S*, and the thermal conductivity *κ* over the temperature range 303–873 K, we can directly correlate the observed 2D‐to‐3D structural transformation with the resulting charge carrier dynamics. Quite interestingly, a series of TE property measurements and evaluations reveal a compelling optimization of TE transport properties driven by the combined effects of enhanced *µ* and balanced *n* due to Sr and Ag co‐doping, simultaneously bringing about the structural transformation.

Figure 6a illustrates the temperature‐dependent *σ* for the five title compounds. Across all compositions, *σ* generally decreases with increasing temperature, a trend characteristic of heavily doped semiconductors governed by acoustic phonon scattering [6, 34]. However, a distinct upturn is observed near 700 K for several compounds, indicating the onset of bipolar transport behavior [35]. By extrapolating the high‐temperature acoustic‐phonon scattering trend, the specific bipolar electrical conductivity contribution can be quantitatively isolated, rationalizing the anomalous conductivity increase above 700 K. The high‐temperature phenomenon is driven by the thermal excitation of minority charge carriers across the bandgap, which simultaneously influences both *σ* and *S*. The highest *σ* values are observed at room temperature, which are 454.8, 426.2, 402.1, 513.5, and 431.7 S/cm for Ca_8.45(3)_Sr_0.55_Zn_4.28(3)_Ag_0.22_Sb_9_, Ca_8.11(3)_Sr_0.89_Zn_4.33(2)_Ag_0.21_Sb_9_, Ca_7.97(3)_Sr_1.03_Zn_4.42(2)_Ag_0.10_Sb_9_, Ca_7.59(2)_Sr_1.41_Zn_4.37(2)_Ag_0.19_Sb_9_, and Ca_7.57(2)_Sr_1.43_Zn_4.27(2)_Ag_0.29_Sb_9_, respectively. Notably, all the Sr‐ and Ag‐co‐doped compounds exhibit significantly higher *σ* values than the ternary pristine phase Ca_9_Zn_4.5_Sb_9_.

**FIGURE 6:**
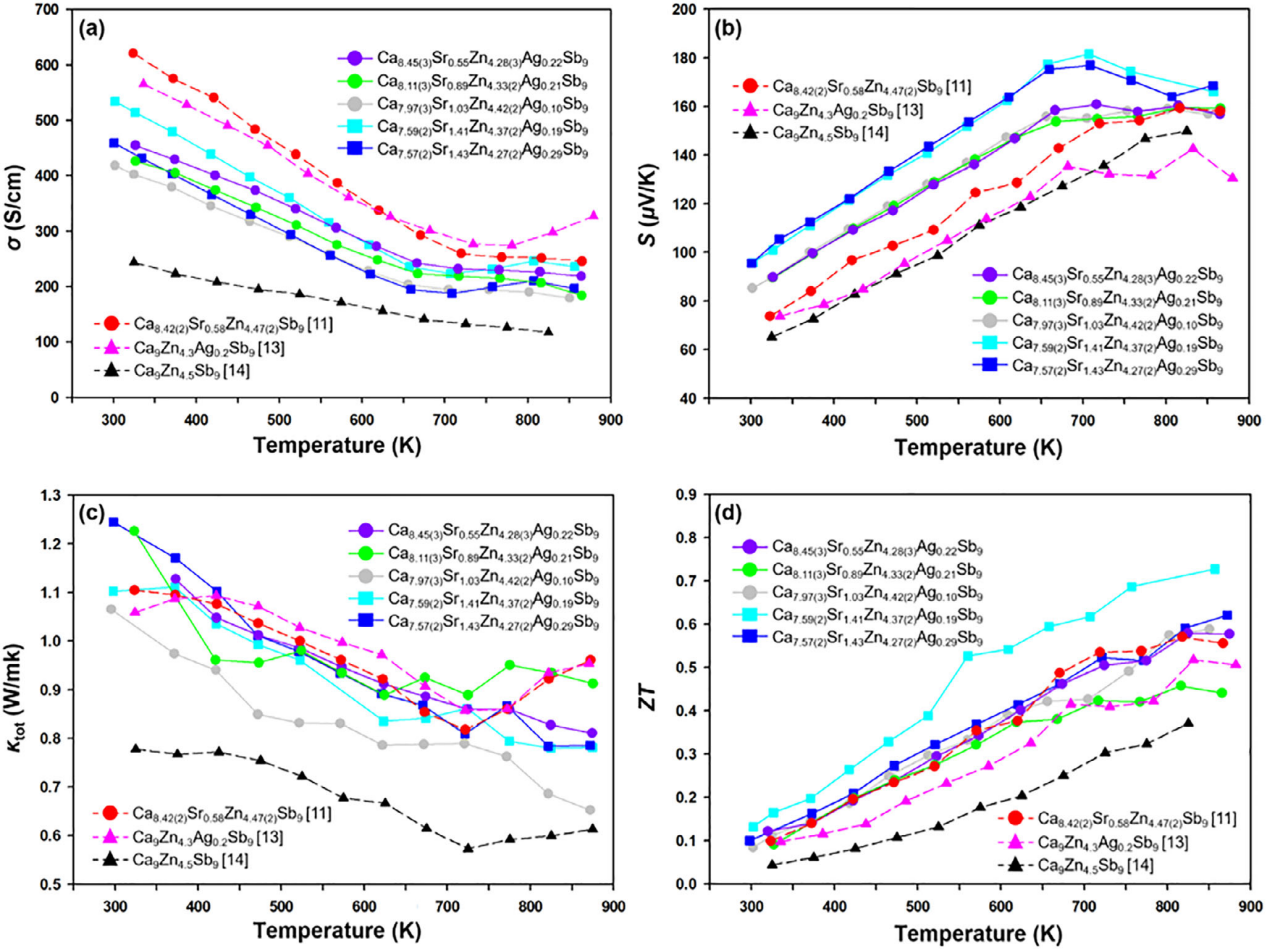
Temperature‐dependent (a) *σ*, (b) *S*, (c) *κ*_tot_, and (d) *ZT* of the five title compounds in the Ca_9‐_ *_x_* Sr*_x_* Zn_4+_ *_y_*_‐_ *_z_* Ag*_z_* Sb_9_ system measured between 303 and 873 K. The TE properties of the three reference compounds: Ca_9_Zn_4.5_Sb_9_, Ca_9_Zn_4.3_Ag_0.2_Sb_9_, and Ca_8.42(2)_Sr_0.58_Zn_4.47(2)_Sb_9_ are also plotted for comparison purposes.

To understand this *σ* enhancement, the *n* and *µ* were thoroughly analyzed and compared in Table 2. In particular, the *n* in the co‐doped compounds is not just influenced by the *p*‐type Ag‐doping, but also affected by the Sr‐doping. The relatively lower electronegativity of Sr than that of Ca suggests an enhanced electron donation effect to the anionic framework in the co‐doped compounds, which may eventually result in a reduced *n*. Interestingly, these two competing effects from the Sr‐ and Ag‐doping can be understood from previous reports: (1) in the Ca_9‐_ *_x_* Sr*_x_* Zn_4.5_Sb_9_ system, the *n* decreased from *ca*. 2.0 × 10^20^ for the parental Ca_9_Zn_4.5_Sb_9_ to *ca*. 1.3 × 10^20^ carriers/cm^3^ for Ca_8.2_Sr_0.8_Zn_4.5_Sb_9_ [11], whereas (2) in the Ca_9_Zn_4.5‐_ *_x_* Ag*_x_* Sb_9_ system, the Ag‐doping increased the *n* from *ca*. 1.9 × 10^20^ for Ca_9_Zn_4.5_Sb_9_ to 2.6 × 10^20^ carriers/cm^3^ for Ca_9_Zn_4.4_Ag_0.1_Sb_9_ [13]. In other words, these results indicate that the Sr‐doping of 0.8 and the Ag‐doping of 0.1 produced comparable but opposite changes in the *n*. Considering that the Sr‐doping level in our title compound Ca_7.97(3)_Sr_1.03_Zn_4.42(2)_Ag_0.10_Sb_9_ is relatively higher than that in the reported Sr‐doped compound, while the Ag‐doping level remains nearly identical, the hole‐reducing effect of the Sr‐doping could be more pronounced than the hole‐generating effect of the Ag‐doping. Therefore, we can claim that the observed Hall effect measurements for the title compounds are consistent with the current interpretation. As shown in Table 2, the *n* decreased from 6.07 × 10^19^ for Ca_9_Zn_4.5_Sb_9_ to 4.88 × 10^19^ carriers/cm^3^ for Ca_7.97(3)_Sr_1.03_Zn_4.42(2)_Ag_0.10_Sb_9_ despite the introduction of the *p*‐type Ag dopant, due to the overwhelming Sr‐doping effect. Moreover, as the Ag content increased from 0.19 to 0.29 while maintaining nearly identical Sr contents in Ca_7.59(2)_Sr_1.41_Zn_4.37(2)_Ag_0.19_Sb_9_ and Ca_7.57(2)_Sr_1.43_Zn_4.27(2)_Ag_0.29_Sb_9_, respectively, the *n* increased by approximately 20% from 4.66 × 10^19^ to 5.60 × 10^19^ carriers/cm^3^.

The temperature‐dependent *S* measurements for the five title compounds are provided in Figure 6b. For all title compounds, *S* values increase steadily with rising temperature before exhibiting a slight downturn beyond *ca*. 700 K. The maximum *S* values for the respective five title compounds reach 160.9, 155.0, 155.1, 181.6, and 177.1 *µV*/K for Ca_8.45(3)_Sr_0.55_Zn_4.28(3)_Ag_0.22_Sb_9_, Ca_8.11(3)_Sr_0.89_Zn_4.33(2)_Ag_0.21_Sb_9_, Ca_7.97(3)_Sr_1.03_Zn_4.42(2)_Ag_0.10_Sb_9_, Ca_7.59(2)_Sr_1.41_Zn_4.37(2)_Ag_0.19_Sb_9_, and Ca_7.57(2)_Sr_1.43_Zn_4.27(2)_Ag_0.29_Sb_9_, respectively. Interestingly, the two fully transformed novel phase Ca_7.59(2)_Sr_1.41_Zn_4.37(2)_Ag_0.19_Sb_9_ and Ca_7.57(2)_Sr_1.43_Zn_4.27(2)_Ag_0.29_Sb_9_ display the largest *S* among these five quinary compounds. This type of *S* enhancement is consistent with the reduced *n* discussed above, but it is also driven by a fundamental change in the band structure. To quantify the correlation to the electronic band structure, *m* ^∗^ was calculated using the measured *n* and *S* values by the Mott relationship: $S=\frac{8\pi^{2}k_{B}^{2}}{3eh^{2}}m^{*}T{\left(\frac{\pi }{3n}\right)}^{\frac{2}{3}}$, where m^*^ is the carrier effective mass, *k*_B_​ is the Boltzmann constant, and *h* is the Planck constant [36]. As illustrated in the Pisarenko plot in Figure S5, the experimental data points do not follow a single theoretical contour line. Instead, as the structure transitions from the 2D layered network to the fully connected 3D framework, the data points distinctly shift across the contours toward higher *m* ^∗^ values. This upward deviation confirms that the remarkable *S* enhancement is not solely due to the reduction in *n*, but is fundamentally driven by a structure‐induced modification of the electronic band structure, increasing *m* ^∗^ from 0.46 *m_e_* ​ to 0.76 *m_e_*. Interestingly, the initial linear rise in *S* should be attributed to this temperature dependence. However, at elevated temperatures (*T* > 723 K), the intrinsic carrier excitation becomes pronounced. Thermal generation of minority carriers introduces bipolar conduction and eventually causes the thermopower to deviate from the expected linear Mott trend quantitatively. The opposing charge signs of electrons and holes counteract each other, ultimately suppressing the overall thermopower. To quantify this bipolar contribution, the bandgap (*E*_g_) was also estimated using the Goldsmid‐Sharp approximation: *E*_g_​ = 2*e*∣*S_max_* ​∣*T_max_* ​. Across all the title compounds, the calculated *E_g_* ​ remains at approximately 0.25–0.26 eV, and these consistent and narrow bandgaps confirm the inherent susceptibility of the title system to minority carrier excitation. Thus, the observed thermopower suppression and bipolar *κ* onset above 700 K for all the title compounds can be nicely rationalized by the narrow bandgaps. The resultant temperature‐dependent power factors *PF* are also provided in Figure S6a.

The total thermal conductivity (*κ*_tot_) for all title compounds was carefully measured, and the results are shown in Figure 6c. Across all compositions, *κ*_tot_ values decrease with increasing temperature, which is consistent with increasing Umklapp (phonon–phonon) scattering in crystalline TE materials, and is also influenced by the temperature‐dependent electronic contribution [37, 38]. The minimum *κ*_tot_ values for the five compounds were recorded as 0.81, 0.89, 0.65, 0.78, and 0.78 W/mK, respectively, for Ca_8.45(3)_Sr_0.55_Zn_4.28(3)_Ag_0.22_Sb_9_, Ca_8.11(3)_Sr_0.89_Zn_4.33(2)_Ag_0.21_Sb_9_, Ca_7.97(3)_Sr_1.03_Zn_4.42(2)_Ag_0.10_Sb_9_, Ca_7.59(2)_Sr_1.41_Zn_4.37(2)_Ag_0.19_Sb_9_, and Ca_7.57(2)_Sr_1.43_Zn_4.27(2)_Ag_0.29_Sb_9_. Interestingly, all the Sr‐ and Ag‐co‐doped quinary compounds exhibit a higher *κ*_tot_ than the ternary parent compound. Typically, substituting Ca^2+^ with the heavier and larger Sr^2+^ is expected to suppress lattice thermal conductivity, *κ*_latt_, via enhanced point‐defect phonon scattering due to mass and size fluctuations [39]. However, this substitution alone hardly explains the changes in electrical transport caused by the framework connectivity and local bonding in the title system. To understand the observed deviation, we must decouple the electronic (*κ*_elec_) and the lattice (*κ*_latt​_) thermal conductivity contributions in the *κ*_tot_ (*κ*_tot_ ​ = *κ*_elec_ ​+ *κ*_latt​_). The *κ*_elec_ can be estimated using the Wiedemann–Franz law: *κ*_elec_ = *LσT*, where the temperature‐dependent Lorenz number (*L*, in 10^−8^ W Ω K^−2^) is approximated by the given equation: $L=1.5+\exp[-\frac{\left|s\right|}{116}]$ [40]. The calculated *κ*_elec_ values near 325 K are 0.28, 0.27, 0.25, 0.32, and 0.26 W/mK for Ca_8.45(3)_Sr_0.55_Zn_4.28(3)_Ag_0.22_Sb_9_, Ca_8.11(3)_Sr_0.89_Zn_4.33(2)_Ag_0.21_Sb_9_, Ca_7.97(3)_Sr_1.03_Zn_4.42(2)_Ag_0.10_Sb_9_, Ca_7.59(2)_Sr_1.41_Zn_4.37(2)_Ag_0.19_Sb_9_, and Ca_7.57(2)_Sr_1.43_Zn_4.27(2)_Ag_0.29_Sb_9_, respectively. It becomes evident that the substantial increase in *σ* drives a proportional rise in *κ*_elec_​, which heavily contributes to the elevated *κ*_tot_​. Furthermore, the *κ*_latt_ obtained by subtracting *κ*_elec_ from *κ*_tot_ is presented in Figure S6b. At intermediate temperatures, *κ*_latt_ roughly follows the 1/*T* dependence characteristic of Umklapp phonon scattering, but at elevated temperatures above 700 K, an upward deviation from this 1/*T* trend is observed. This type of deviation quantifies the bipolar thermal conductivity, which arises directly from the thermal excitation of electron–hole pairs across the narrow bandgap calculated earlier.

Interestingly, recent studies show that thermal transport in complex crystals cannot simply be understood by the mass‐disorder scattering alone. Particularly relevant to the present Zintl system, diffuson‐driven transport in Eu_14_MgAs_11_ challenges a simple relationship between atomic mass and *κ*_latt_. In addition, studies for Cs_2_AgBiBr_6_, o‐CsCu_5_S_3_, and Tl_9_SbTe_6_ further show that strong anharmonicity, higher‐order phonon scattering, and wave‐like interbranch coherence can jointly determine *κ*_tot_ [41]. In these studies, coherence provides an additional heat‐transfer channel rather than an additional scattering mechanism. Moreover, anharmonicity does not invariably lower conductivity, but temperature‐induced phonon hardening can enhance particle‐like transport in BaZrO_3_ and YbFe_4_Sb_12_ [42]. Structural directionality is also important, as demonstrated by orientation‐controlled heat flow in Sb_2_S_3_ and coherence‐enhanced interlayer transport in CsCu_4_Se_3_ [43]. These studies provide broader context in terms of thermal transport in our present Sr‐induced reconstruction from a 2D layered network to a 3D framework. In addition to substitutional disorder, the redistribution of Zn site occupancies and the formation of Zn─Sb bonds can modify the vibrational spectrum and phonon‐scattering processes. The disappearance of the refined Zn2/Zn3 split‐site configuration in the fully transformed phase establishes a change in site occupancy, but does not by itself demonstrate reduced phonon scattering. Accordingly, the current *κ*_tot_ trends are interpreted as the combined outcome of electronic heat transport, substitutional disorder, and reconstructed framework connectivity, rather than mass fluctuations alone. Although our experimental investigation provides a robust macroscopic evaluation of the *κ*_latt_, quantifying the microscopic anharmonicity, higher‐order phonon scattering, and coherence contributions would require advanced finite‐temperature atomistic simulations, such as DFT‐based phonon Boltzmann transport equation calculations or molecular dynamics, which remain an important direction for future computational studies beyond the scope of our current experimental work.

As displayed in Figure 6d, the dimensionless TE figure‐of‐merit *ZT* was calculated as a function of temperature. All the quinary title compounds exhibit a continuous increase in *ZT* with temperature, peaking near 873 K. Crucially, the quantitative impact of the bipolar effect becomes highly apparent in this high‐temperature regime. Although the bipolar excitation provides a slight enhancement to *σ*, the simultaneous rise in bipolar *κ* and the suppression of *S* dominate the overall transport behavior. As a result of this combination, the narrow 0.25 eV bandgap acts as the fundamental constraint that limits the maximum achievable *ZT* above 700 K. Despite this observation, compared with the parent ternary compound, these co‐doped quinary compounds demonstrate significantly enhanced TE performance across the entire measured temperature range. Among the investigated series, the fully transformed quinary Ca_7.59(2)_Sr_1.41_Zn_4.37(2)_Ag_0.19_Sb_9_ achieves the highest *ZT* value of *ca*. 0.73 at 873 K, which is a remarkable 2‐fold improvement over the ternary parent Ca_9_Zn_4.5_Sb_9_. This exceptional performance is achieved because the particular composition simultaneously possesses the largest *S* and a considerably high *σ*. As discussed earlier, the fully connected 3D anionic framework provides additional conduction pathways that enhance mobility, while heavy Sr substitution increases m*. Thus, the observed perfect synergy leads to the highest *PF* and, consequently, the maximum *ZT*. The rest of the four co‐doped quinary compounds also display strong high‐temperature performance, with maximum *ZT* values ranging from 0.45 to 0.62. To evaluate the effectiveness of this synergistic co‐doping strategy, the maximum *ZT* value of the fully transformed compound Ca_7.59(2)_Sr_1.41_Zn_4.37(2)_Ag_0.19_Sb_9_ was compared with those of previously reported analogs in the A_9_M_4+_ *_x_* Sb_9_ (A = Ca, Sr, Eu, Yb; M = Zn, Cu, Ag) system. As provided in Table S5, the maximum *ZT* of 0.73 for the quinary compound is competitive enough among this family of analogs.

The temperature‐dependent weighted mobility (*µ*_w_​) and TE quality factor (*B*) were also calculated and the results are illustrated in Figure S7. The *µ*_w_, which reflects the intrinsic electronic transport quality, increases dramatically from 17.3 cm^2^/Vs in the parental ternary Ca_9_Zn_4.56_Sb_9_ to 66.9 cm^2^/Vs in the fully transformed quinary Ca_7.59(2)_Sr_1.41_Zn_4.37(2)_Ag_0.19_Sb_9_ at room temperature. In addition, analysis of *B* reveals that the observed structural transformation from the 2D to the 3D phase fundamentally upgrades the intrinsic TE properties of the title compounds, increasing *B* from 0.128 in the parent ternary phase to a maximum of 0.328 in the fully transformed quinary phase. This significant enhancement in TE properties nicely coincides with the maximum *ZT* of 0.73, while higher Ag doping (*z* = 0.29) slightly degrades *B*.

Lastly, a further analysis of the compositional trends reveals a fascinating transport mechanism. In the intermediate structure regime of the 2D anionic layers in the title system, *σ* generally decreases with increasing Sr content and increases with increasing Ag content, while variations in *κ*_tot_ reflect changes in both electronic and lattice heat transport. However, these simple compositional trends break down in the fully transformed 3D phase. In particular, the fully transformed 3D structures exhibit higher *ZT* values than their intermediate counterparts because they achieve simultaneous enhancements in both *σ* and *S*. This behavior deviates from the conventional inverse trade‐off relationship between *σ* and *S* [44, 45]. Although the *κ*_tot_ increases slightly in the intermediate phase due to the heightened electronic contribution, the exponential improvement in the *PF* of the fully transformed phase heavily outweighs the thermal penalty. Finally, it is shown that the remarkable enhancement in *ZT* is not merely a compositional doping effect, but is fundamentally rooted in the optimized charge transport characteristics induced by the Sr‐induced structural transformation.

### 2.4 Machine Learning Validation

#### 2.4.1 Dataset Context and Model Robustness

To validate the temperature‐dependent TE properties of the title Ca_9‐_ *_x_* Sr*_x_* Zn_4+_ *_y_*_‐_ *_z_* Ag*_z_* Sb_9_ system using a series of ML processes, a hybrid transfer ML method combining CrabNet and XGBoost was developed. The three ML models for ​*σ*, *S*, and ​*κ*_tot_ were trained using a strategic combination of (1) the generalized Starry dataset [46] for broad chemical feature extraction and (2) the highly curated experimentally studied thermoelectric materials (ESTM) dataset [47] for high‐fidelity regression. Comprehensive details regarding dataset curation are provided in Section S2, and elemental frequencies, structural diversity, and property distributions are provided in Figures S8–S11. Furthermore, quantitative analyses of the learned latent space, rigorous 10‐fold cross‐validation metrics, feature sensitivity evaluations, learning curves, and detailed error analyses at 873 K were used to assess the robustness, physical accuracy, systematic bias, and limitations of these ML models (Section S2, Figures S12–S16).

#### 2.4.2 Feature Representation and Predictive Power

To verify that the CrabNet model [18, 48] learned the fundamental physical rules of TE transport behavior rather than merely memorizing the training data, we visualized its internal feature representations using t‐distributed stochastic neighbor embedding (t‐SNE) [49]. The global latent space projections of log_10_​*σ*, *S*, and log_10_​*κ*_tot​_ reveal smooth, continuous property gradients across the title Ca_9‐_ *_x_* Sr*_x_* Zn_4+_ *_y_*_‐_ *_z_* Ag*_z_* Sb_9_ system (Figure 7a,c,e). Interestingly, the *S* projection clearly separates *n*‐type and *p*‐type materials into distinct physical domains, while high‐performance regions across all three properties are grouped together with minimal outliers. On the other hand, isolating the targeted A_9_M_4+_ *_x_* Pn_9_‐type Zintl phases reveals that these phases are grouped together rather than randomly scattered in the latent space, as shown in Figure 7b,d, and f. Specifically, these are localized at the top‐center edge for log_10_​*σ*, the top‐left edge for *S*, and the bottom‐right edge for log_10_​*κ*_tot_, placing them directly in the particular composition of optimal TE properties. Given that the ML models successfully identified a well‐defined, physically sound neighborhood for the known A_9_M_4+_ *_x_* Pn_9_ family, we can be highly confident in their ability to accurately predict the transport properties of our novel Sr‐ and Ag‐co‐doped Ca_9‐_ *_x_* Sr*_x_* Zn_4+_ *_y_*_‐_ *_z_* Ag*_z_* Sb_9_ system.

**FIGURE 7:**
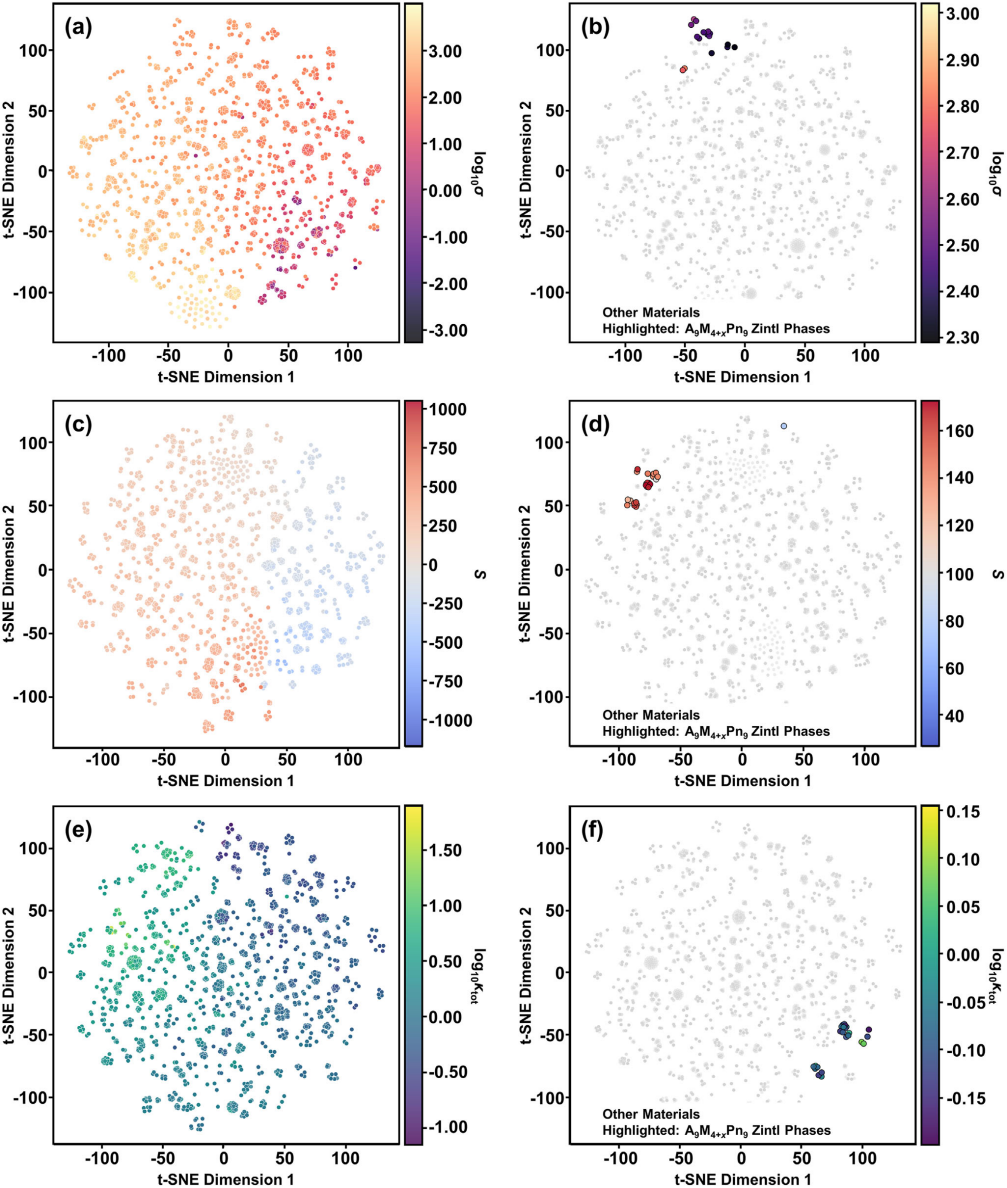
t‐SNE visualizations of the CrabNet‐derived latent space for log_10_ *σ*, *S*, and log_10_ *κ*_tot_ of (a,c,e) the global property gradients and (b,d,f) the isolated A_9_M_4+_ *_x_* Pn_9_ families. The targeted A_9_M_4+_ *_x_* Pn_9_ Zintl phases are grouped together within the optimal high‐performance regions of chemical space.

To quantitatively evaluate the predictive power of the CrabNet‐extracted features, we trained XGBoost regressors [50] to map these latent representations to temperature‐dependent TE properties (Figure 8). The resulting models generalize robustly across the entire title system, achieving strong test‐set coefficients of determination (*R* ^2^) of 0.814, 0.860, and 0.874 for log_10_​*σ*, *S*, and log_10_ *κ*_tot​_, respectively. Parity plots (See Figure 8a,d,g) confirm that the predicted values closely track the experimental data along the ideal fit line. The log_10_​*σ* model exhibits the most scattered data (RMSE = 0.433, MAE = 0.241), particularly within the low‐conductivity regime, while the *S* model (RMSE = 78.862 *µV*/K, MAE = 35.942 *µV*/K) shows occasional outliers in the mid‐to‐high *p*‐type range. On the other hand, the log_10_​*κ*_tot​_ model yields the highest accuracy with minimal scatter (RMSE = 0.128, MAE = 0.070). In Figure 8b,e,h, heteroscedasticity checks reveal that while outlier magnitudes can increase at the physical extremes, such as low conductivities or highly negative *n*‐type Seebeck values, the residual errors remain evenly distributed around zero. Furthermore, the error density histograms for all three properties are highly symmetric Gaussian distributions centered exactly at zero, with no skewed tails, confirming the absence of systematic bias (See Figure 8c,f,i). Ultimately, these metrics prove that the hybrid ML method provides highly reliable, unbiased predictions, perfectly setting the stage for evaluating our novel quinary Ca_9‐_ *_x_* Sr*_x_* Zn_4+_ *_y_*_‐_ *_z_* Ag*_z_* Sb_9_ systems.

**FIGURE 8:**
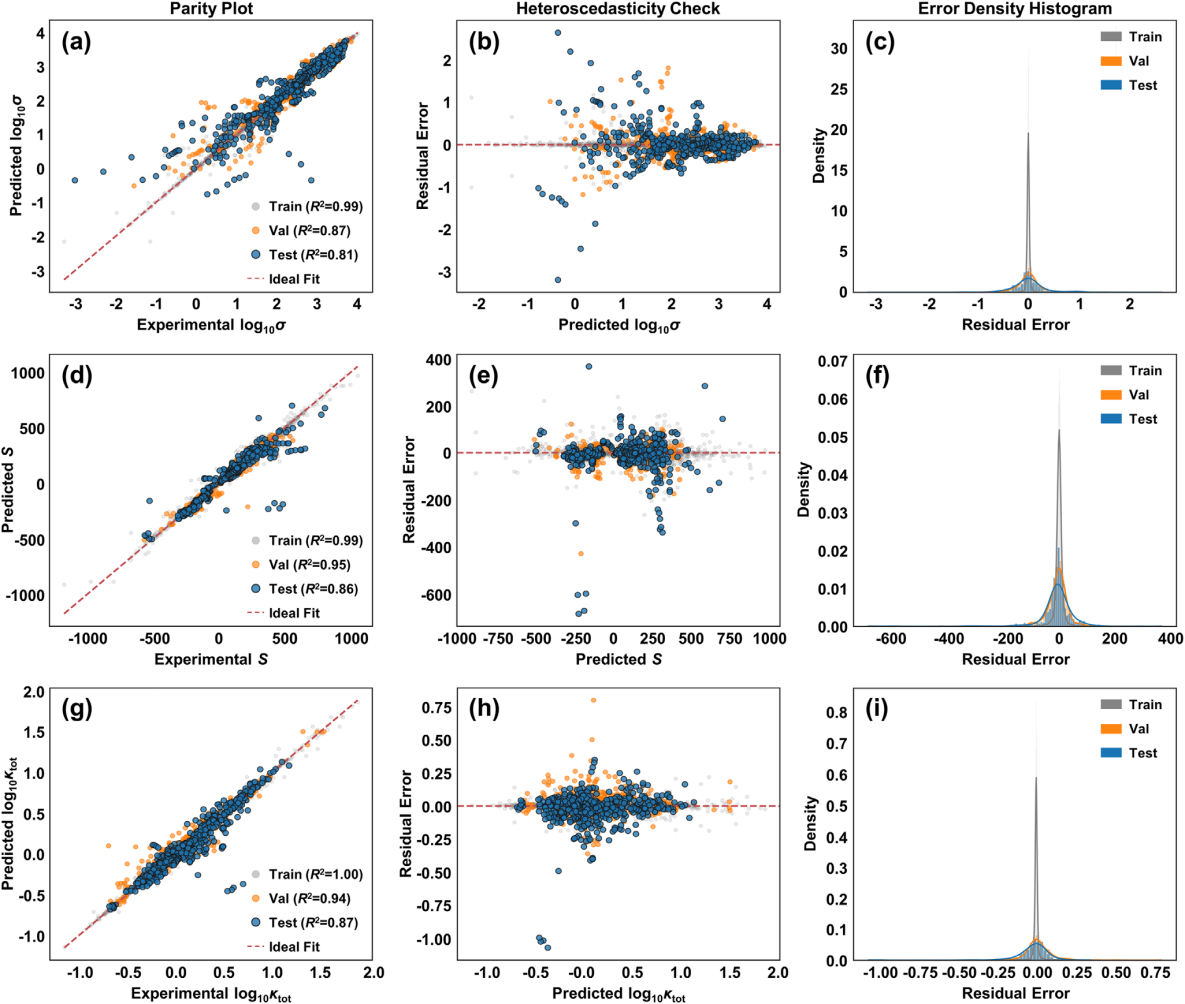
Parity plots, heteroscedasticity checks, and error density histograms for the three XGBoost regression ML models predicting (a–c) log_10_ *σ*, (d–f) *S*, and (g–i) log_10_ *κ*_tot_. The three ML models show robust generalization, high *R* ^2^ values, and highly symmetrical error distributions, indicating no systematic bias.

#### 2.4.3 Predictive Validation and Compositional Mapping

The three ML models trained by our CrabNet‐XGBoost hybrid‐transfer method were finally deployed to predict temperature‐dependent TE properties for a total of eight experimentally determined compounds: one ternary, two quaternary, and five quinary compounds. Through this evaluation, we were also able to validate the predictive capability of our hybrid method and its potential utility as a compositional trend screening tool. As shown in Figure 9, these ML models successfully capture the fundamental temperature dependencies by accurately predicting the continuous decrease in *σ* and *κ*_tot_, as well as the initial rise in *S*. However, since ML models often struggle to capture intrinsic multi‐band excitations without explicit physical descriptors [51], the models hardly capture the high‐temperature bipolar transport effects in TE properties, specifically the slight upturn in *σ* and downturn in *S* as experimentally observed above 700 K. Despite these kinds of ML deviations at elevated temperatures, our models overall demonstrate exceptional accuracy in compositional ranking. For instance, ML models correctly identify the quinary Ca_7.57_Sr_1.43_Zn_4.27_Ag_0.29_Sb_9_ as possessing the highest *S*. Interestingly, the quaternary Ca_8.42_Sr_0.58_Zn_4.47_Sb_9_ is predicted to have the second‐highest *S*, located just above the next best quinary Ca_7.97_Sr_1.03_Zn_4.42_Ag_0.10_Sb_9_. Furthermore, these models accurately capture the phenomenon that the ternary parent compound generally exhibits lower *κ*_tot_​ than most of the quinary compounds, with the notable exception of the two heavily doped best title compounds. Finally, the dimensionless *ZT* was calculated directly from the individually predicted three TE properties to evaluate the overall predicted performance of the ML models. These ML models successfully identify the experimentally observed best‐performing compounds, predicting a maximum *ZT* of 1.14 for Ca_7.59_Sr_1.41_Zn_4.37_Ag_0.19_Sb_9_, followed closely by 1.07 for Ca_7.57_Sr_1.43_Zn_4.27_Ag_0.29_Sb_9_. Although these predicted maximum *ZT* values overestimate the experimental values due to the overpredicted *σ*, the identification of the best composition supports the usefulness of the ML model for selecting potential candidates. However, because the errors from the separately predicted *σ*, *S*, and *κ*_tot_ propagate into *ZT*, the quantitative interpretation of the predicted maxima is limited. This result clearly demonstrates that our CrabNet‐XGBoost hybrid ML method serves as a reliable directional compass for accelerating the discovery of high‐performance TE materials prior to the comprehensive experimental exploration. Finally, it should be noted that composition‐based ML models hardly reflect the sample processing, microstructure, site occupancies, or defect configurations in the synthesized products. These excluded variables may contribute to the conductivity discrepancy, but the current ML analysis cannot isolate these effects. Therefore, the ML predictions in this work are exploited for relative compositional trend assessment and exploratory screening, rather than quantitatively accurate TE property prediction or independent validation of the structural transformation mechanism.

**FIGURE 9:**
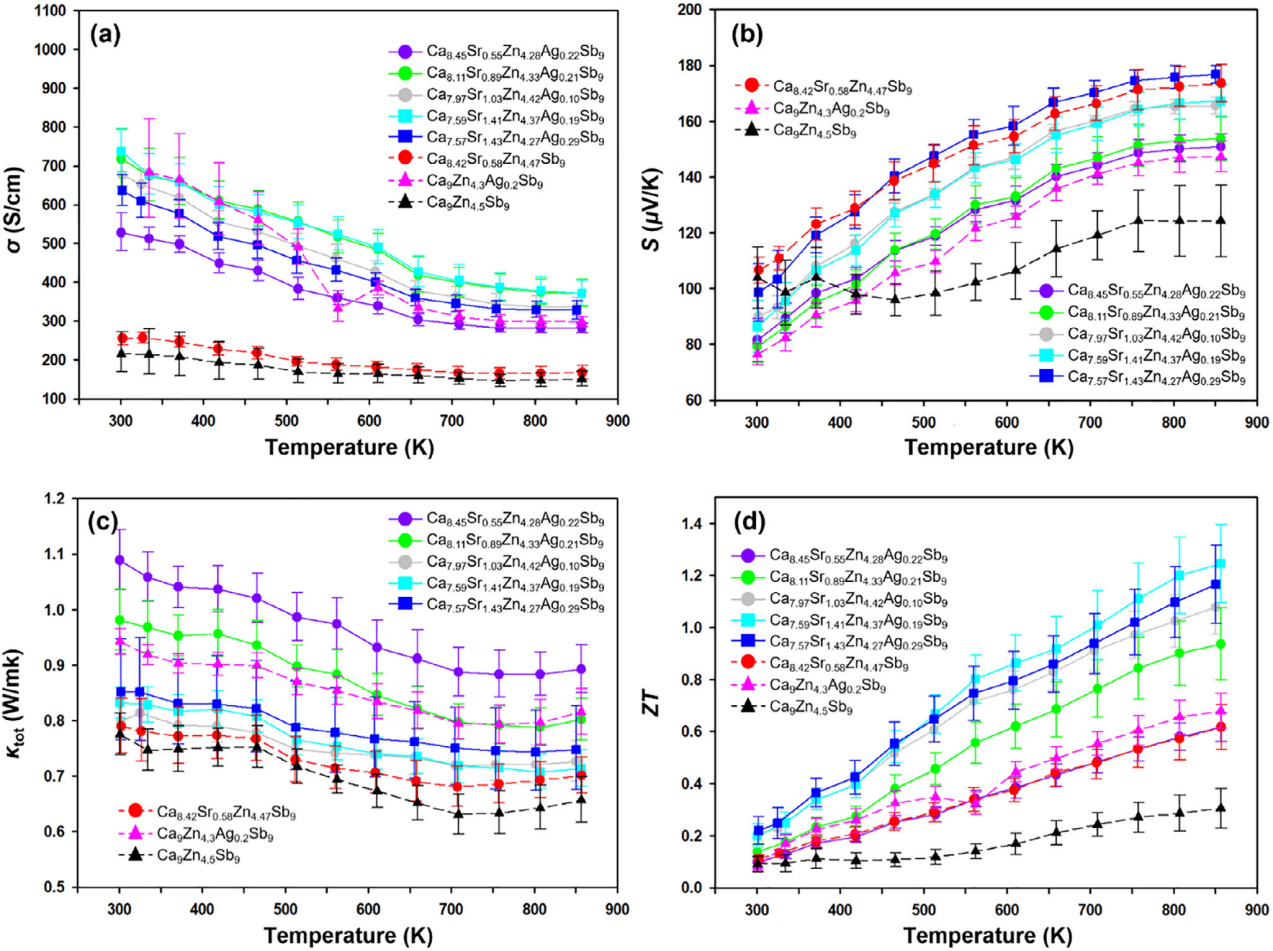
ML‐predicted temperature‐dependent TE properties of (a) *σ*, (b) *S*, (c) *κ*_tot_, and (d) *ZT* for the synthesized Ca_9‐_ *_x_* Sr*_x_* Zn_4+_ *_y_*_‐_ *_z_* Ag*_z_* Sb_9_ compounds. The models accurately capture the temperature‐dependent trends and preserve the relative compositional ranking among the highest‐performing compositions. Error bars for *σ*, *S*, and *κ*_tot_, and the uncertainty bounds for *ZT* propagated from the individual property models are also provided.

The 2D contour maps of the predicted TE properties were generated at 873 K to visualize the composition‐dependent predictions in the title Ca_9‐_ *_x_* Sr*_x_* Zn_4+_ *_y_*_‐_ *_z_* Ag*_z_* Sb_9_ system as displayed in Figure 10. The predicted *σ* reaches its maximum value near *x* = 1.0 and *y* = 0.25, initially increasing with Ag content before declining upon further addition (See Figure 10a), while the *S* demonstrates a classic inverse relationship, peaking precisely in regions of lower *σ* as provided in Figure 10b. In addition, the 2D map of *κ*_tot_​ confirms that the lowest thermal transport occurs in the Sr‐rich region. However, this trend does not independently establish enhanced phonon scattering since *κ*_tot_ includes both electronic and lattice contributions, and the composition‐based ML predictions hardly resolve microscopic transport mechanisms. The overall combination of these competing TE properties is nicely illustrated in the dimensionless *ZT* contour map in Figure 10d. The experimental data points were also overlaid to compare the spatial accuracy of the ML models. Although the absolute experimental *ZT* values are not in perfect agreement with the background prediction contour maps due to the over‐prediction of the *σ* model, the *ZT* contour map successfully identifies the broader high *ZT* region. More interestingly, the ML model predicts the global maximum of *ZT* at approximately *x* = 1.55 and *y* = 0.32, which is close to the experimentally observed value for our best‐performing compound having *x* = 1.5 and *y* = 0.2. This result indicates that although we were able to successfully capture a highly optimized composition via the experimental investigation in the current work, the CrabNet‐XGBoost hybrid method can also be exploited to predict a promising candidate and provide a clear direction for future experimental investigation and property optimization. It can be noted that the ML‐predicted high‐performance regions beyond the experimentally verified samples should be carefully considered for future investigation due to its uncertainty. Therefore, the maps provide exploratory compositional guidance rather than real validation of structural or transport mechanisms.

**FIGURE 10:**
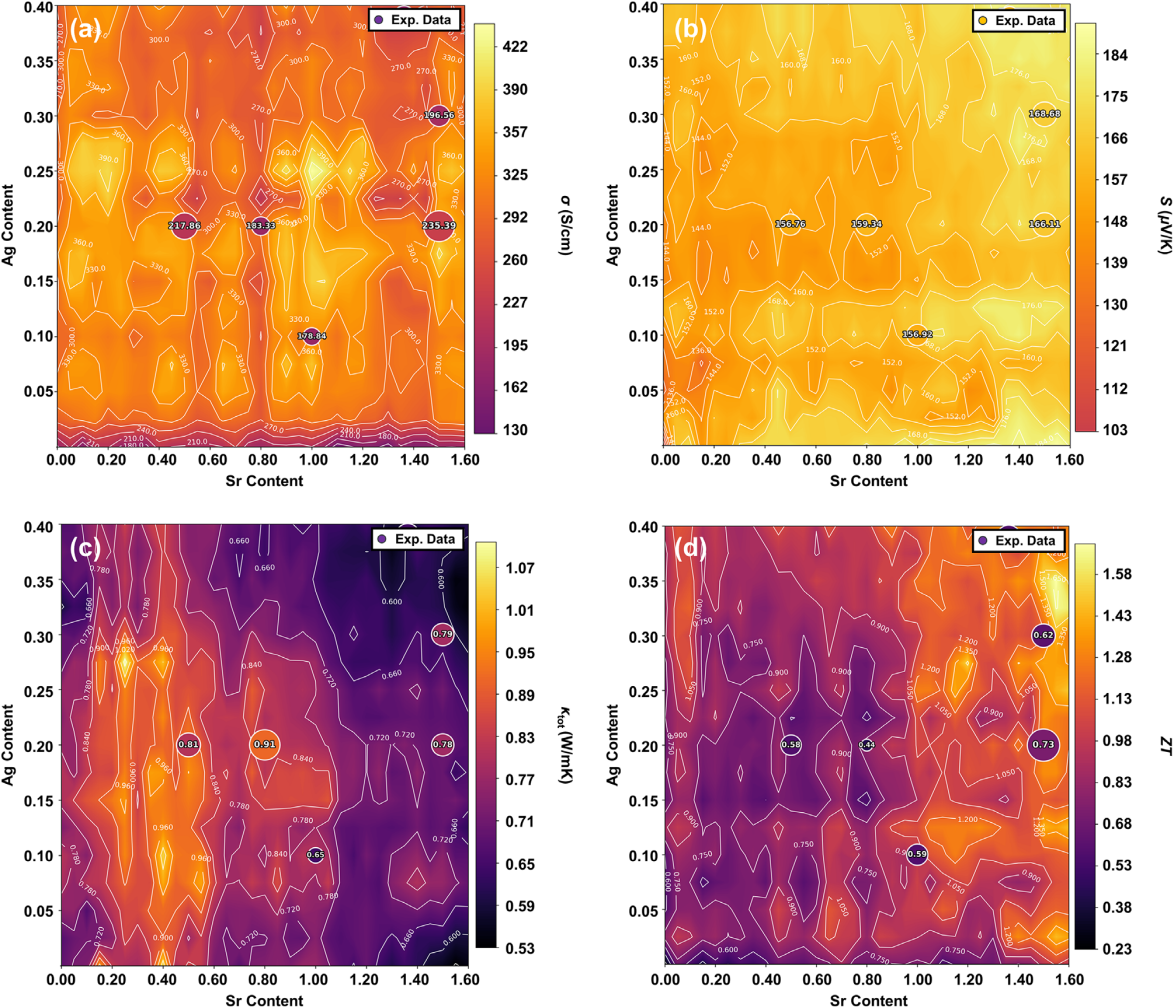
2D compositional contour maps of predicted (a) *σ*, (b) *S*, (c) *κ*_tot_, and (d) *ZT* as functions of the Sr and Ag contents at 873 K. The models capture the fundamental transport trade‐offs and identify a predicted global *ZT* maximum at approximately *x* = 1.55 and *y* = 0.32.

## 3 Conclusion

This work shows that synergistic Sr‐ and Ag‐co‐doping in the title Ca_9‐_ *_x_* Sr*_x_* Zn_4+_ *_y_*_‐_ *_z_* Ag*_z_* Sb_9_ system provides an effective strategy for simultaneously tuning structural topology and TE transport properties within the A_9_M_4+x_Pn_9_ Zintl series. Combined crystallographic, electronic structure, TE property, and hybrid ML analyses reveal that substitution of the relatively larger Sr^2+^ cation progressively introduces steric strain into the crystal structure, eventually driving complete Zn migration and inducing a topological reconstruction from the parent 2D zigzag‐shaped anionic layers to a fully connected 3D anionic framework. This structural evolution is not just a crystallographic modification, but the key origin of the enhanced transport behavior observed in the fully transformed phase. Therefore, the reconstructed 3D framework creates more effective charge‐transport pathways, while Ag incorporation and the accompanying electronic‐structure modification reduce bond polarity and preserve a favorable electronic environment near *E*_F_. As a result, the fully transformed phase exhibits substantially improved *µ* and *σ*, while maintaining a sufficiently large *S*. In particular, the quinary title compound Ca_7.59(2)_Sr_1.41_Zn_4.37(2)_Ag_0.19_Sb_9_ achieves a maximum *ZT* of *ca*. 0.73 at 873 K, representing a significant enhancement over the ternary parent compound. Overall, these results establish steric‐pressure‐driven topological reconstruction as a practical design principle for optimizing complex Zintl TE materials beyond conventional carrier concentration tuning. Moreover, the CrabNet‐XGBoost hybrid transfer‐learning approach successfully reproduces the experimentally observed compositional trends and identifies the high‐performance region in composition space, highlighting the value of integrating data‐driven validation with experimental and theoretical investigation. Overall, this work demonstrates that structural topology engineering, supported by thorough ML validation, provides a powerful pathway to advance high‐performance Zintl TE materials.

## 4 Experimental Section

### 4.1 Synthesis

Single crystals of the five title compounds in the Ca_9‐_ *_x_* Sr*_x_* Zn_4+_ *_y_*_‐_ *_z_* Ag*_z_* Sb_9_ (0.55(3) ≤ *x* ≤ 1.43(2); 0.50(3) ≤ *y* ≤ 0.56(2); 0.10(2) ≤ *z* ≤ 0.29(2)) system were synthesized by the molten Pb‐flux method. The well‐grown bar/needle‐shaped single crystals were successfully obtained, and the remaining trace of Pb flux on the crystals was removed via hydrogen peroxide etching. For TE property measurements, bulk polycrystalline samples were prepared from stoichiometric elemental powders by high‐energy ball milling, followed by densification using a uniaxial hot‐press method. Detailed synthetic parameters, including exact molar ratios, heating profiles, and pressing conditions, are provided in Section S1.

### 4.2 Structural Characterization

Phase purity and detailed crystal structures of the title compounds were characterized using PXRD and SXRD. Elemental compositions and distribution maps, as well as high‐temperature thermal stabilities, were confirmed using energy‐dispersive X‐ray spectroscopy (EDS) and thermogravimetric analysis (TGA), respectively. To evaluate TE performance, *σ*, *S*, and thermal diffusivity were measured over the temperature range 303–873 K. These measurements were supplemented by room‐temperature Hall effect measurements to determine *n* and *µ*. Full instrumental details, measurement configurations, and crystallographic data are available in Section S1, Table 1, Tables S1 and S2.

### 4.3 Electronic Structure Calculations

DFT calculations were performed using the TB‐LMTO‐ASA method [24, 25, 26] to investigate the overall electronic structures and bonding characteristics of the title Ca_9‐_ *_x_* Sr*_x_* Zn_4+_ *_y_*_‐_ *_z_* Ag*_z_* Sb_9_ system. Analyses of DOS, COHP, and ELF were conducted on the three hypothetical structural models with idealized compositions [24, 25, 26, 52]. To accurately model partial occupancies and mixed substitutions, the symmetry of the experimentally determined crystal structures was reduced, utilizing structural parameters derived directly from SXRD refinements. Comprehensive computational parameters, including Wigner–Seitz (WS) radii, basis sets, and *k*‐point meshes, are detailed in the Section S1.

### 4.4 ML Methods and Predictive Modeling

To validate our experimental findings and further explore the compositional space of the title system, a predictive ML method was performed. Standard graph neural networks (GNNs) [53] often require precise 3D crystallographic inputs and struggle to incorporate continuous thermodynamic state variables, such as temperature. To overcome these limitations and bridge static chemistry with dynamic testing conditions, we implemented a hybrid transfer learning architecture that couples deep compositional representation learning with gradient‐boosted regression. The core predictive engine operates via the following two‐stage procedure. In the first stage, CrabNet [18, 48] serves as the primary feature extractor, and by processing chemical formulas with a transformer‐based self‐attention mechanism, it captures complex chemical interactions without requiring explicit 3D inputs. To address experimental data scarcity, the CrabNet was pre‐trained on a large‐scale dataset from Starry [46] to learn fundamental chemical rules, and then applied to a high‐fidelity experimental dataset that combines publicly available ESTM [47] data with our proprietary in‐house A_9_M_4+_ *_x_* Pn_9_ Zintl phase measurements. A 512‐dimensional latent vector, acting as a dense mathematical fingerprint of the material, was extracted from this network. In the second stage, the static chemical embedding was concatenated with the scalar temperature variable and fed to an XGBoost regressor [19, 50] to model nonlinear physical dependencies. Then, we implemented a rigorous 10‐fold cross‐validation protocol combined with strict composition‐based splitting to ensure that our performance metrics reflect true predictive capability rather than data leakage. This protocol evaluates prediction for held‐out compositions but does not necessarily exclude related chemical families or nearby doping levels from training. After this evaluation, the fully trained models were utilized to predict and validate the TE characteristics of the synthesized compositions. Comprehensive details on data preprocessing, hyperparameter optimization, and computational reproducibility are provided in Section S3.

## Author Contributions

**Siyeon Kim**: data curation, investigation, validation, formal analysis, visualization, writing – original draft; **Aziz Ahmed**: software, data curation, investigation, validation, writing – original draft; **Juwon Lee**: investigation, formal analysis, visualization; **Jihyun Lee**: data curation, formal analysis; **Ji Hee Pi**: data curation, formal analysis; **Kyu Hyoung Lee**: funding acquisition, resources; **Kang Min Ok**: data curation, resources; **Tae‐Soo You**: conceptualization, methodology, formal analysis, supervision, funding acquisition, project administration, resources, writing – original draft, writing – review & editing.

## Conflicts of Interest

The authors declare no conflicts of interest.

## Supporting information




**Supporting File**: advs78519‐sup‐0001‐SuppMat.docx.

## Data Availability

The data that support the findings of this study are available from the corresponding author upon reasonable request.
