# Supplementary material for: Steric‐Pressure‐Driven Topological Reconstruction for High‐Performance Zintl Phase Thermoelectrics with Machine‐Learning Validation
Source: Adv Sci (Weinh). 2026 Oct 26:e78519. Online ahead of print. doi: 10.1002/advs.78519 (PMC13710726; doi:10.1002/advs.78519)
Supplement: Supplementary file 1 [file ADVS-9999-e78519-s001.docx]

**Supporting Information**

**Steric-Pressure-Driven Topological Reconstruction for High-Performance Zintl Phase Thermoelectrics with Machine-Learning Validation**

Siyeon Kim,^1^ Aziz Ahmed,^1^ Juwon Lee,^1^ Jihyun Lee,^2^ Ji Hee Pi,^3^ Kyu Hyoung Lee,^3^ Kang Min Ok,^2^

and Tae-Soo You^1*^

^1^Department of Chemistry, Chungbuk National University, Cheongju, Chungbuk 28644, Republic of Korea

^2^Department of Chemistry, Sogang University, Seoul 04107, Republic of Korea

^3^Department of Materials Science and Engineering, Yonsei University, Seoul 03722, Republic of Korea.

**CORRESPONDING AUTHOR**

**Name**: Tae-Soo You

**E-mail**: tsyou@chungbuk.ac.kr

**Phone**: +82 (43) 261-2282

**S.1 Experimental Methodology**

**Synthesis.** All sample preparation procedures were carried out in an Ar-filled glovebox to avoid oxidation of the air-sensitive elements. The starting materials were purchased from Alfa Aesar, including Ca (shot, 99.5 %), Sr (granules, 99 %), Zn (shot, 99.99 %), Sb (shot, 99.999 %), and Pb (granules, 99.99 %), while Ag (wire, 99.99 %) was purchased from Nilaco. Prior to use, the tanned surfaces of Ca and Sr elements were cleaned with a metal brush to remove surface oxide layers. Single crystals of the five title compounds were synthesized using a molten Pb-flux method. The starting reactant mixtures were placed in alumina crucibles, together with an excess amount of Pb metal serving as the inactive metal flux. The nominal molar ratios were as follows: Ca:Sr:Zn:Ag:Sb:Pb = 8.5:0.5:4.3:0.2:9:45, 8.2:0.8:4.3:0.2:9:45, 8:1:4.4:0.1:9:45, 8:1:4.3:0.2:9:45, and 8:1:4.2:0.3:9:45 for the experimentally obtained compositions of Ca_8.45(3)_Sr_0.55_Zn_4.28(3)_Ag_0.22_Sb_9_, Ca_8.11(3)_Sr_0.89_Zn_4.33(2)_Ag_0.21_Sb_9_, Ca_7.97(3)_Sr_1.03_Zn_4.42(2)_Ag_0.10_Sb_9,_ Ca_7.59(2)_Sr_1.41_Zn_4.37(2)_Ag_0.19_Sb_9_, and Ca_7.57(2)_Sr_1.43_Zn_4.27(2)_Ag_0.29_Sb_9_, respectively. Each alumina crucible containing the reaction mixtures was placed in a fused-silica tube, which was subsequently evacuated and sealed under vacuum to prevent oxidation during the high-temperature reaction. The sealed tubes were heated to 1323 K at 200 K/h, maintained for 24 h, and slowly cooled to 873 K at 5 K/h over 90 h. The tubes were then removed at 873 K and immediately centrifuged for 3 min to separate the molten Pb flux. As a result, well-formed needle/bar-shaped single crystals were obtained as shown in Figure S17. Residual Pb was removed by immersing the product in a diluted 35% hydrogen peroxide solution for two days. For TE measurements, bulk samples were prepared by ball milling followed by hot pressing. Starting materials were loaded into a stainless-steel milling container with two 0.5-inch and two 0.25-inch stainless-steel balls. The mixtures were ball-milled for 2 h 30 min using a SPEX 8000M mill, pausing every 30 min to scrape and remix the powders. The obtained powder was transferred into a 12.5 mm graphite die and heated to 623 K under a uniaxial pressure of 60 MPa for 1 h 45 min, held for 2 h, then increased to 1073 K over 2 h 15 min and maintained at 1073 K for 2 h before cooling to room temperature.

**X-ray Diffraction Analysis.** PXRD patterns were collected using the Bruker D8 diffractometer with Cu K*α*_1_ radiation (*λ* = 1.54059 Å), and the results are shown in Figure S18. Measurements were carried out for 30 min per sample with a step size of 0.02° over the angular range of 15° ≤ 2*θ* ≤ 85°. The phase purity of each sample was confirmed by comparing the experimentally obtained PXRD patterns with the simulated PXRD pattern generated from the SXRD refinement results of Ca_7.59(2)_Sr_1.41_Zn_4.37(2)_Ag_0.19_Sb_9_. All experimentally obtained PXRD patterns showed good agreement with the simulated patterns, confirming that the title compounds successfully crystallize in both the intermediate Ca_9_Mn_4_Bi_9_-type phase and the fully transformed Ca_7.6_Sr_1.4_Zn_4.4_Ag_0.2_Sb_9_-type phase. SXRD data were collected at room temperature using a Bruker D8 QUEST diffractometer with Mo Kα1 radiation (*λ* = 0.71073 Å). After preliminary examination of several well-grown single crystals, the best-quality crystal was selected for full data collection. Data collection was performed using the Bruker APEX3 program,[S1] while data reduction, integration, and unit cell parameter refinements were carried out using the SAINT program.[S2] Semi-empirical absorption corrections based on equivalents were applied using the SADABS program.[S3] All data were consistent with the orthorhombic *Pbam* space group (No. 55). The crystal structures were refined by full-matrix least-squares methods on *F*², including the atomic positions with anisotropic displacement parameters (ADPs), extinction coefficients, and mixed occupancies of the mixed ratios of Ca and Sr, and Zn and Ag. In the final stage of refinement, the STRUCTURE TIDY program[S4] was applied to standardize the atomic coordinates. Detailed crystallographic parameters, including atomic coordinates with ADP values and selected interatomic distances, are summarized in Table 1, Tables S1, and S2. The complete crystallographic data have been deposited at the Cambridge Crystallographic Data Centre (CCDC) and can be obtained free of charge from www.ccdc.cam.ac.uk/data_request/cif. The deposition numbers are as follow: CCDC-2579949 for Ca_8.45(3)_Sr_0.55_Zn_4.28(3)_Ag_0.22_Sb_9_, CCDC-2579952 for Ca_8.11(3)_Sr_0.89_Zn_4.33(2)_Ag_0.21_Sb_9_, CCDC-2579951 for Ca_7.97(3)_Sr_1.03_Zn_4.42(2)_Ag_0.10_Sb_9_, CCDC-2579953 for Ca_7.59(2)_Sr_1.41_Zn_4.37(2)_Ag_0.19_Sb_9_, and CCDC-2579950 for Ca_7.57(2)_Sr_1.43_Zn_4.27(2)_Ag_0.29_Sb_9._

**Electronic Structure Calculations.** A series of DFT calculations was carried out using the TB-LMTO-ASA method[26-28] to understand the electronic structures of the title compounds. Three hypothetical structural models with idealized compositions of Ca_9_Zn_4.5_Sb_9_, Ca_8_SrZn_4.25_Ag_0.25_Sb_9_, and Ca_7.5_Sr_1.5_Zn_4.25_Ag_0.25_Sb_9_ were designed for the calculations. The resultant DOS, COHP, and ELF diagrams were thoroughly analyzed to investigate the overall electronic structure and bonding characteristics of the title system. To design the partial occupancies and mixed substitutions, the symmetry of these models was reduced from the experimentally determined space group *Pbam* (No. 55) to its subgroup *Pm* (No. 6). The lattice parameters and atomic coordinates were taken from the SXRD refinement results of the corresponding compounds: Ca_9_Zn_4.48(2)_Sb_9_, Ca_8.11(3)_Sr_0.89_Zn_4.33(2)_Ag_0.21_Sb_9_ and Ca_7.59(2)_Sr_1.41_Zn_4.37(2)_Ag_0.19_Sb_9_, which are close to the compositions of the hypothetical models. Detailed structural information for the three models is provided in Supporting Information Table S4. Within the ASA scheme,[26] the entire unit cell was filled with overlapping WS spheres.[28] Relativistic effects were included using a scalar relativistic approximation, except for spin–orbit coupling.[S5] Each WS sphere was assumed to contain a spherically symmetric potential, and a combined correction was applied to account for the overlapping regions.[S6] The radii of the WS spheres were automatically optimized to provide the best approximation to the full potential. The WS radii used for the three models are as follows, Ca: 1.921−2.189 Å, Zn: 1.345−1.513 Å, Sb: 1.637−1.941 Å for Ca_9_Zn_4.5_Sb_9_; Ca: 1.950-2.246 Å, Sr: 2.204 Å, Zn: 1.404−1.511 Å, Ag: 1.460 Å, Sb: 1.660−1.947 Å for Ca_8_SrZn_4.25_Ag_0.25_Sb_9_; and Ca: 1.946-2.255 Å, Sr: 2.207 Å, Zn: 1.420-1.509 Å, Ag: 1.487 Å, Sb: 1.668-1.945 Å for Ca_7.5_Sr_1.5_Zn_4.25_Ag_0.25_Sb­_9_. The basis sets included 4s, 4p and 3d orbitals for Ca; 5s, 5p, 4d, and 4f orbitals for Sr; 4s, 4p, and 3d orbitals for Zn; 5s, 5p, 4d and 4f orbitals for Ag; and 5s, 5p, 5d, and 4f orbitals for Sb. The Löwdin downfolding technique[S7] was applied for the Ca 4p, Sr 5p and 4f, Ag 4f, and Sb 5d and 4f orbitals. The self-consistent charge density was obtained using 320 irreducible *k*-points in the Brillouin zone for all three models.

**Thermogravimetric (TGA) Analysis.** The thermal stabilities of the title compounds were examined by TGA using a HITACHI STA200. Approximately 15 mg of pulverized sample was placed on an alumina pan and heated to 1173 K at 10 K/min under a continuous flow of N_2_ gas. After the measurement, the sample was naturally cooled to room temperature. All compounds remained thermally stable up to approximately 900 K, as shown in Supporting Information Figure S19.

**Electrical and Thermal Transport Measurements.** Bar-shaped samples (3 mm × 3 mm × 10 mm) with densities > 92% of the theoretical density were used for electrical transport property measurements. The longest direction of each bar-shaped sample was aligned with the measurement direction. The *σ* and *S* were simultaneously measured over the temperature range of 303 to 873 K under a He atmosphere using a ULVAC-RIKO ZEM-3 instrument system. Thermal diffusivity of the five disk-shaped samples was measured under an inert gas atmosphere in the temperature range of 303 to 873 K using a Netzsch LFA 467 HyperFlash instrument based on the flash diffusivity method. In this technique, the front surface of the disk-shaped sample was irradiated with a short laser burst, and the temperature rise at the rear surface was detected by an IR detector. The thermal conductivity *κ* was evaluated using the relation *κ* = *DC*_p_*ρ*, where *D* is the thermal diffusivity, *C*_p_ is heat capacity, and *ρ* is the density. In this study, the Dulong−Petit approximation (3*R*/atom, where *R* is the gas constant) was adopted for *C*_p_.[S8,S9] The *κ*_tot_ was considered to consist of the *κ*_latt_ and the *κ*_elec_. The electronic contribution *κ*_elec_ was estimated using the Wiedemann-Franz law (*κ*_elec_ = *LσT*), where *L* is the temperature-dependent Lorenz number.[42,S10] The *L* value was determined using the single-parabolic band (SPB) model based on the measured temperature-dependent *S*. Accordingly, *κ*_latt_ was obtained from the following equation: *κ*_latt_ = *κ*_tot_ − *κ*_elec_.

**EDS Analysis.** Elemental compositions and distribution maps were analyzed by EDS using an ULTRA Plus field-emission scanning electron microscope system operated at an accelerating voltage of 20 kV. Several well-shaped single crystals obtained from the molten Pb-flux method were carefully selected and mounted on an aluminum puck using double-sided conductive carbon tape under an Ar atmosphere. Detailed EDS analysis and elemental mapping results are provided in Supporting Information Figure S20.

**Hall Measurements.** Hall measurements were conducted at room temperature in a magnetic field of 0.55 T using a van der Pauw four-point probe configuration (HMS-3000, Ecopia). The disk-shaped samples had a diameter of 12.5 mm and a thickness of 1.0 mm. The Hall voltage (*V*_H_) is expressed as *V*_H_ = *IB*/*qnd* (*I*: the current flowing through the sample, *B*: the magnetic field strength, *q*: the carrier charge, *n*: the number of mobile charge carriers, *d*: the thickness of the sample).[S11,S12] The *μ* was determined using *μ* = 1/*qnR*, where *R* is the electrical resistivity.[S11,S12]

**S.2 Machine Learning Validation Details**

**Dataset Curation and Structural Diversity.** To computationally validate the observed temperature-dependent TE properties of the title compounds, a hybrid transfer learning framework was developed using two distinct materials databases: the generalized Starry dataset[48] and the highly curated ESTM dataset.[49] As visualized in the periodic table heatmaps (Figure S8), the elemental frequencies highlight the complementary nature of these datasets. The Starry dataset provides a broad chemical background, while the ESTM dataset offers a more focused chemical space. Crucially, both datasets contain robust representations of our targeted Zintl elements, including Ca, Sr, Zn, Ag, and Sb. Since the Starry dataset is broader but inherently noisier, chemical formulas from both databases were combined exclusively during the initial representation learning phase to extract deep, composition-derived elemental embeddings using CrabNet.[19] Subsequently, only the high-fidelity ESTM dataset was utilized to train the final XGBoost[20] regression models. Beyond elemental composition, the structural diversity of the training data dictates the ability of the model to interpret complex crystallographic phenomena. Figure S9 illustrates the distribution of distinct material families within the Starry (2,765 compounds) and ESTM (893 compounds) datasets. In particular, the Starry dataset provides a massive, generalized structural foundation, dominated by IV-VI and V-VI chalcogenides, skutterudites, and oxides (Figure S9a). Conversely, the curated ESTM dataset presents a more specialized structural distribution tailored for high-fidelity regression (Figure S9b). Notably, the ESTM dataset exhibits a significantly higher concentration of Zintl phases (over 16% combined). More importantly, the ESTM dataset explicitly includes the A_9_M_4+_*_x_*Pn_9_ sub-family (3.0%), which directly corresponds to our target Ca_9-_*_x_*Sr*_x_*Zn_4+_*_y_*_-_*_z_*Ag*_z_*Sb_9_ system.

**Property Distributions and Temperature Dependence.** The property distributions across the training datasets were examined to ensure the ML algorithms interpolate within a physically realistic domain (Figure S10). The *σ* reveals a stark contrast in data quality: the broad Starry dataset exhibits an irregular distribution, whereas the curated ESTM dataset forms a well-defined Gaussian curve that more closely represents refined semiconducting behavior. The *S* distributions display a distinct bimodal nature corresponding to *n*-type and *p*-type carriers. Furthermore, the *κ*_tot_ highlights the superiority of the ESTM dataset for high-performance TE materials, as its distribution shifts noticeably lower. Since our Ca_9-_*_x_*Sr*_x_*Zn_4+_*_y_*_-_*_z_*Ag*_z_*Sb_9_ system utilizes a complex cationic structure and a 2D-to-3D topological reconstruction to scatter phonons effectively, its experimental *κ*_tot_ values naturally align with the favorable lower end of this ESTM distribution. To validate that the ML models capture the dynamic physics of charge and phonon transport, the temperature-dependent TE properties were analyzed across the datasets (Figure S11). For *σ*, the curated ESTM dataset displays a general decrease in *σ* with increasing temperature, characteristic of degenerate semiconducting behavior. The *S* trajectories further separate *p*-type and *n*-type materials as temperature rises. The A_9_M_4+_*_x_*Pn_9_ compounds in the ESTM dataset follow an ideal trajectory with *S* steadily increasing to peak values around 200 *μ*V/K before exhibiting a distinct high-temperature rollover, perfectly capturing the bipolar effect. Finally, *κ*_tot_ decreases monotonically with temperature across both datasets, illustrating classic Umklapp phonon scattering.

**Latent Space Cross-Correlation.** Crucially, while the Ca_9-_*_x_*Sr*_x_*Zn_4+_*_y_*_-_*_z_*Ag*_z_*Sb_9_ system was entirely absent from the training data, quantitative analysis of the latent space reveals that the models learned a highly transferable,[22] property-specific encoding of fundamental solid-state physics as illustrated in Figure S12. The model successfully isolates the electron-donating sublattice from the covalently bonded framework, as evidenced by the weak cross-correlation between the alkaline-earth metals and the Zn/Ag-Sb polyanionic framework (*r* = 0.227-0.437). For instance, the learned similarity between Ca and Sr is strongly correlated in the log_10_*σ* model (*r* = 0.765), reflecting their shared +2 valence, but drops precipitously in the log_10_*κ*_tot_ model (*r* = 0.224). This demonstrates the autonomous recognition of the model of their distinct atomic masses and phonon scattering cross sections. Similarly, the weakened correlation between Zn and Ag in the *S* model (*r* = 0.724) confirms the capacity of the model to anticipate the valence-driven hole doping effects of Ag^+^ substitution.

**Cross-Validation and Feature Sensitivity.** A 10-fold cross-validation[S13] was performed to rigorously validate the generalizability of our ML method, and the results were displayed in Figure S13. A strict composition-based split was enforced across all folds to prevent data leakage. The models exhibited exceptional stability and robust predictive power across all folds. The log_10_*κ*_tot_ model demonstrated the highest consistency, yielding a mean cross-validated *R*^2^ of 0.925. The *S* model similarly maintained high accuracy, averaging an *R*^2^ of 0.899. These values summarize performance under the specified composition-grouped protocol, rather than transfer to entirely unseen chemical families. While the log_10_*σ* model inherently faces greater experimental variance, it still achieved a strong mean *R*^2^ of 0.839. To evaluate the informational density of the CrabNet-derived latent space, we performed a feature sensitivity analysis as shown in Figure S14. For both *S* and log_10_*κ*_tot_, predictive performance maximized when utilizing the full suite of 513 features. On the other hand, the log_10_*σ* model achieved its peak *R*^2^ using only the top 10 most important features. However, increasing the feature count from 10 to the full 513 resulted in only a small decrease in predictive accuracy. To maintain a unified ML procedure, we elected to utilize the complete 513-feature set for all final models. This analysis compares subsets of the existing latent representation and does not establish that the descriptors are globally optimal.

**Learning Curve Analysis.** Learning curves were constructed by increasing the training-set size from approximately 10% to 100% of the available training data to assess the dependence of predictive performance on data availability (Figure S15). For log_10_*σ*, increasing the training-set size from 429 to 4299 improved the test *R*^2^ from 0.658 to 0.814 and reduced the test MAE from 0.318 to 0.241. Most of the improvement in *R*^2^ occurred within the first 30% of the training data, followed by smaller, nonmonotonic changes, indicating diminishing gains over the upper training-size range. In contrast, the *S* model showed substantial improvement at the largest training sizes: increasing the training-set size from 3478 to 4348 improved the test *R*^2^ from 0.759 to 0.860 and reduced the MAE from 43.206 to 35.942 *μ*V/K. This behavior suggests that predictive performance may benefit from additional relevant training data, although improvement beyond the investigated range is not established. For log_10_κ_tot_, the test *R*^2^ increased from 0.851 at 434 training samples to 0.874 at 4347 samples, with fluctuations across intermediate sizes, while the MAE decreased from 0.098 to 0.070. Thus, although the thermal-conductivity model exhibited relatively limited changes in *R*^2^, additional data generally reduced its absolute prediction error in logarithmic space. Overall, these learning curves show property-dependent benefits from increasing training-set size under the present evaluation protocol.

**Quantitative Error Analysis at 873 K.** To rigorously evaluate the predictive accuracy of the model at the temperature of maximum TE performance at 873 K, a quantitative error analysis was conducted across all synthesized compositions (Figure S16).[7] The CrabNet-XGBoost hybrid method demonstrates remarkable precision in predicting *S* and *κ*_tot_. For the quinary Sr/Ag co-doped compounds, the absolute relative error for *S* remains predominantly below 5%. At the same time, *κ*_tot_ errors are generally well-constrained between 3.8% and 23%, while *σ* is systematically overpredicted across the compositional matrix. Given that *σ* is highly sensitive to microstructural factors like grain boundaries and defect scattering that are not explicitly encoded in an ideal-crystal chemical formula, this magnitude deviation is a common and expected limitation of global ML models. As a result, this overestimation directly propagates into the calculated *ZT*. However, despite these absolute magnitude deviations, the models perfectly preserve the relative compositional ranking, confirming that this kind of hybrid ML method serves as a highly robust screening tool.

**S.3 ML Methodology**

**ML Data Curation and Preprocessing.** The predictive models were trained using a curated dataset aggregated from distinct sources. Foundational chemical feature learning was conducted using a large-scale, heterogeneous Starry/ESTM dataset.[48,49] For precise regression of TE properties, a high-fidelity dataset was constructed by combining the ESTM dataset[7,49] with our proprietary A_9_M_4+_*_x_*Pn_9_ Zintl phase dataset. All raw chemical formulas were parsed and validated using the pymatgen library,[S14] and entries with invalid stoichiometries or missing targets were removed. Since *σ* and *κ*_tot_ span several orders of magnitude, these two properties were represented as log_10_*σ* and log_10_*κ*_tot_, respectively, where *σ* is expressed in S/cm and *κ*_tot_ in W/m·K. The *S*, on the other hand, is given directly in *μ*V/K. To resolve the lack of a temperature factor in composition-only models when faced with multiple temperature-dependent measurements, a "Median Aggregation" strategy[S15] was implemented. The target value for each unique chemical formula was defined as the median of its temperature-dependent measurements, providing a statistically robust representation that is insensitive to high-temperature measurement artifacts or outliers.

**Hybrid Model Architecture.** The primary feature extractor CrabNet[18,S16] employs a self-attention mechanism that evaluates the fractional abundances of constituent elements to learn complex bonding environments. A transfer learning strategy was used to overcome data scarcity. The model was pre-trained on the Starry dataset to learn elemental similarities, valency trends, and stable stoichiometric ratios. To prevent data contamination, any chemical formulas present in the experimental ESTM dataset were strictly purged from the Starry dataset prior to pre-training. During training on the experimental dataset, a custom initialization patch was applied to the Stochastic Weight Averaging optimizer in PyTorch[S17] for compatibility. Upon convergence, network weights were frozen, and a 512-dimensional latent vector was extracted from the penultimate layer. This 512-dimensional static embedding was concatenated with the scalar temperature variable to reintroduce thermodynamic context, resulting in a total of a 513-dimensional feature space. This vector was fed into an XGBoost regressor, leveraging the capacity of CrabNet for stoichiometric rules and the efficiency of XGBoost in modeling non-linear physical dependencies.

**Validation Strategy and Inference Protocol.** A rigorous 10-fold cross-validation protocol with strict composition-based splitting was implemented. The dataset was grouped exclusively by unique chemical formulas and divided into ten equal segments. Then, the model was trained on nine portions and validated against the tenth, repeating until every segment served as an independent test set. This procedure ensured that measurements at different temperatures for the same grouped composition did not appear in both training and test sets. It did not, by itself, exclude related compositions or entire material families. Final performance metrics were calculated as the average of these ten iterations. The hyperparameters of the XGBoost regression head were optimized using the Optuna Bayesian optimization framework[20,S18] to minimize the Root Mean Squared Error (RMSE). To ensure absolute scientific reproducibility, a global random seed was enforced across all stochastic operations, including NumPy array permutations,[S19] PyTorch weight initializations,[S17] and CuDNN deterministic backends.[S20] During the embedding extraction phase, an evaluation state was enforced to turn off stochastic network behaviors like Dropout,[S17,S21] guaranteeing that the generated chemical embedding for any formula remains mathematically identical across all inference runs.

| **TABLE S1.** Atomic coordinates and equivalent isotropic displacement parameters (*U*_eq_*^a^*) from the SXRD refinements for the Ca_9-_*_x_*Sr*_x_*Zn_4+_*_y_*_-_*_z_*Ag*_z_*Sb_9_ (0.55(3) ≤ *x* ≤ 1.43(2); 0.50(3) ≤ *y* ≤ 0.56(2); 0.10(2) ≤ *z* ≤ 0.29(2)) system | | | | | | |
| --- | --- | --- | --- | --- | --- | --- |
| Atom | Wyckoff site | Occupation | *x* | *y* | *z* | *U*_eq_*^a^* (Å^2^) |
| Ca_8.45(3)_Sr_0.55_Zn_4.28(3)_Ag_0.22_Sb_9_ | | | | | | |
| Ca1/Sr1*^b^* | 2*a* | 0.88(2)/0.12 | 0 | 0 | 0 | 0.0147(8) |
| Ca2 | 4*h* | 1 | 0.3628(2) | 0.3601(2) | 1/2 | 0.0139(3) |
| Ca3 | 4*h* | 1 | 0.0613(2) | 0.4092(2) | 1/2 | 0.0108(3) |
| Ca4 | 4*h* | 1 | 0.1244(2) | 0.2389(1) | 1/2 | 0.0155(4) |
| Ca5/Sr5 *^b^* | 4*g* | 0.78(2)/0.22 | 0.2903(2) | 0.1014(2) | 0 | 0.0156(5) |
| Zn1 | 4*g* | 1 | 0.2216(2) | 0.4532(2) | 0 | 0.0158(2) |
| Zn2 | 4*g* | 0.92(1) | 0.3803(2) | 0.2604(1) | 0 | 0.0179(3) |
| Zn3 | 4*g* | 0.08(1) | 0.4800(11) | 0.2244(5) | 0 | 0.0179(3) |
| Zn4/Ag | 4*h* | 0.14(2)/0.11 | 0.0992(4) | 0.1007(2) | 1/2 | 0.0267(14) |
| Sb1 | 2*c* | 1 | 0 | 1/2 | 0 | 0.0096(2) |
| Sb2 | 4*h* | 1 | 0.3060(2) | 0.5013(1) | 1/2 | 0.0115(2) |
| Sb3 | 4*h* | 1 | 0.3786(2) | 0.1949(1) | 1/2 | 0.0105(2) |
| Sb4 | 4*g* | 1 | 0.0403(1) | 0.1484(1) | 0 | 0.0089(2) |
| Sb5 | 4*g* | 1 | 0.1904(2) | 0.3327(2) | 0 | 0.0075(2) |
| Ca_8.11(3)_Sr_0.89_Zn_4.33(2)_Ag_0.21_Sb_9_ | | | | | | |
| Ca1/Sr1*^b^* | 2*a* | 0.73(2)/0.27 | 0 | 0 | 0 | 0.0241(7) |
| Ca2 | 4*h* | 1 | 0.3670(2) | 0.3457(1) | 1/2 | 0.0164(3) |
| Ca3 | 4*h* | 1 | 0.0644(2) | 0.4088(2) | 1/2 | 0.0129(3) |
| Ca4 | 4*h* | 1 | 0.1445(2) | 0.2328(1) | 1/2 | 0.0150(3) |
| Ca5/Sr5 *^b^* | 4*g* | 0.69(2)/0.31 | 0.2937(1) | 0.0962(1) | 0 | 0.0161(4) |
| Zn1 | 4*g* | 1 | 0.2208(2) | 0.4493(2) | 0 | 0.0232(2) |
| Zn2 | 4*g* | 0.09(1) | 0.3884(8) | 0.2604(5) | 0 | 0.0211(2) |
| Zn3 | 4*g* | 0.91(1) | 0.4847(2) | 0.2221(1) | 0 | 0.0211(2) |
| Zn4/Ag | 4*h* | 0.16(2)/0.11 | 0.0908(2) | 0.0946(2) | 1/2 | 0.0254(11) |
| Sb1 | 2*c* | 1 | 0 | 1/2 | 0 | 0.0122(2) |
| Sb2 | 4*h* | 1 | 0.3080(1) | 0.4945(2) | 1/2 | 0.0147(2) |
| Sb3 | 4*h* | 1 | 0.3839(1) | 0.1926(1) | 1/2 | 0.0120(2) |
| Sb4 | 4*g* | 1 | 0.0460(2) | 0.1502(1) | 0 | 0.0118(2) |
| Sb5 | 4*g* | 1 | 0.1900(2) | 0.3292(1) | 0 | 0.0100(2) |
| Ca_7.97(3)_Sr_1.03_Zn_4.42(2)_Ag_0.10_Sb_9_ | | | | | | |
| Ca1/Sr1*^b^* | 2*a* | 0.68(2)/0.32 | 0 | 0 | 0 | 0.0230(6) |
| Ca2 | 4*h* | 1 | 0.3673(2) | 0.3456(1) | 1/2 | 0.0144(3) |
| Ca3 | 4*h* | 1 | 0.0645(2) | 0.4087(1) | 1/2 | 0.0117(3) |
| Ca4 | 4*h* | 1 | 0.1448(2) | 0.2328(2) | 1/2 | 0.0132(3) |
| Ca5/Sr5 *^b^* | 4*g* | 0.64(2)/0.36 | 0.2938(2) | 0.0962(2) | 0 | 0.0150(4) |
| Zn1 | 4*g* | 1 | 0.2207(2) | 0.4492(2) | 0 | 0.0226(2) |
| Zn2 | 4*g* | 0.08(1) | 0.3886(9) | 0.2595(6) | 0 | 0.0203(2) |
| Zn3 | 4*g* | 0.92(1) | 0.4850(2) | 0.2222(2) | 0 | 0.0203(2) |
| Zn4/Ag | 4*h* | 0.21(2)/0.05 | 0.0907(3) | 0.0948(2) | 1/2 | 0.0248(12) |
| Sb1 | 2*c* | 1 | 0 | 1/2 | 0 | 0.0116(2) |
| Sb2 | 4*h* | 1 | 0.3079(2) | 0.4944(2) | 1/2 | 0.0134(2) |
| Sb3 | 4*h* | 1 | 0.3843(1) | 0.1927(1) | 1/2 | 0.0109(2) |
| Sb4 | 4*g* | 1 | 0.0462(1) | 0.1503(1) | 0 | 0.0110(2) |
| Sb5 | 4*g* | 1 | 0.1900(1) | 0.3291(2) | 0 | 0.0091(2) |
| Ca_7.59(2)_Sr_1.41_Zn_4.37(2)_Ag_0.19_Sb_9_ | | | | | | |
| Ca1/Sr1*^b^* | 2*a* | 0.58(2)/0.42 | 0 | 0 | 0 | 0.0235(6) |
| Ca2/Sr2 *^b^* | 4*h* | 0.95(2)/0.05 | 0.3671(2) | 0.3448(2) | 1/2 | 0.0147(5) |
| Ca3 | 4*h* | 1 | 0.0643(2) | 0.4087(1) | 1/2 | 0.0114(3) |
| Ca4 | 4*h* | 1 | 0.1456(2) | 0.2326(2) | 1/2 | 0.0124(3) |
| Ca5/Sr5 *^b^* | 4*g* | 0.56(2)/0.44 | 0.2938(1) | 0.0960(1) | 0 | 0.0150(4) |
| Zn1 | 4*g* | 1 | 0.2203(2) | 0.4488(1) | 0 | 0.0226(2) |
| Zn2 | 4*g* | 1 | 0.4851(1) | 0.2220(2) | 0 | 0.0211(2) |
| Zn3/Ag | 4*h* | 0.19(2)/0.09 | 0.0913(2) | 0.0948(2) | 1/2 | 0.0228(10) |
| Sb1 | 2*c* | 1 | 0 | 1/2 | 0 | 0.0113(2) |
| Sb2 | 4*h* | 1 | 0.3077(1) | 0.4936(1) | 1/2 | 0.0131(2) |
| Sb3 | 4*h* | 1 | 0.3845(1) | 0.1927(1) | 1/2 | 0.0106(2) |
| Sb4 | 4*g* | 1 | 0.0462(1) | 0.1508(1) | 0 | 0.0108(2) |
| Sb5 | 4*g* | 1 | 0.1895(2) | 0.3289(1) | 0 | 0.0089(2) |
| Ca_7.57(2)_Sr_1.43_Zn_4.27(2)_Ag_0.29_Sb_9_ | | | | | | |
| Ca1/Sr1*^b^* | 2*a* | 0.56(2)/0.44 | 0 | 0 | 0 | 0.0242(6) |
| Ca2/Sr2 *^b^* | 4*h* | 0.95(1)/0.05 | 0.3671(2) | 0.3446(1) | 1/2 | 0.0154(6) |
| Ca3 | 4*h* | 1 | 0.0644(2) | 0.4088(1) | 1/2 | 0.0126(3) |
| Ca4 | 4*h* | 1 | 0.1457(2) | 0.2326(1) | 1/2 | 0.0136(3) |
| Ca5/Sr5 *^b^* | 4*g* | 0.55(2)/0.45 | 0.2939(2) | 0.0959(1) | 0 | 0.0159(4) |
| Zn1 | 4*g* | 1 | 0.2203(2) | 0.4487(2) | 0 | 0.0241(2) |

| Zn2 | 4*g* | 1 | 0.4851(2) | 0.2218(1) | 0 | 0.0219(2) |
| --- | --- | --- | --- | --- | --- | --- |
| Zn3/Ag | 4*h* | 0.14(2)/0.14 | 0.0917(2) | 0.0949(2) | 1/2 | 0.0228(10) |
| Sb1 | 2*c* | 1 | 0 | 1/2 | 0 | 0.0127(2) |
| Sb2 | 4*h* | 1 | 0.3076(1) | 0.4933(1) | 1/2 | 0.0147(2) |
| Sb3 | 4*h* | 1 | 0.3844(2) | 0.1927(1) | 1/2 | 0.0119(2) |
| Sb4 | 4*g* | 1 | 0.0460(1) | 0.1510(2) | 0 | 0.0122(2) |
| Sb5 | 4*g* | 1 | 0.1893(1) | 0.3289(1) | 0 | 0.0101(2) |
| *^a^U*_eq_ is defined as one-third of the trace of the orthogonalized *U_ij_* tensor.  *^b^*For the Ca/Sr mixed sites, the numbering for Sr follows that of Ca for convenience. | | | | | | |

| **TABLE S2.** Selected interatomic distances (Å) for the Ca_9-_*_x_*Sr*_x_*Zn_4+_*_y_*_-_*_z_*Ag*_z_*Sb_9_ (0.55(3) ≤ *x* ≤ 1.43(2); 0.50(3) ≤ *y* ≤ 0.56(2); 0.10(2) ≤ *z* ≤ 0.29(2)) system | | | | | | |  |
| --- | --- | --- | --- | --- | --- | --- | --- |
| Atomic pair | Interatomic distance (Å) | | | Atomic pair | Interatomic distance (Å) | |  |
|  | (Intermediate Ca_9_Mn_4_Bi_9_-type) | | |  | (Ca_7.6_Sr_1.4_Zn_4.4_Ag_0.2_Sb_9_-type) | |  |
|  | Ca_8.45(3)_Sr_0.55_Zn_4.28(3)_Ag_0.22_Sb_9_ | Ca_8.11(3)_Sr_0.89_Zn_4.33(2)_Ag_0.21_Sb_9_ | Ca_7.97(3)_Sr_1.03_Zn_4.42(2)_Ag_0.10_Sb_9_ |  | Ca_7.59(2)_Sr_1.41_Zn_4.37(2)_Ag_0.19_Sb_9_ | Ca_7.57(2)_Sr_1.43_Zn_4.27(2)_Ag_0.29_Sb_9_ |  |
| Ca1/Sr1*^a^*-Sb2 (× 4) | 3.338(2) | 3.346(1) | 3.350(1) | Ca1/Sr1*^a^*-Sb2 (× 4) | 3.358(1) | 3.360(2) |  |
| Ca1/Sr1*^a^*-Sb4 (× 2) | 3.297(2) | 3.327(2) | 3.330(2) | Ca1/Sr1*^a^*-Sb4 (× 2) | 3.347(2) | 3.353(2) |  |
| Ca2-Sb2 | 3.181(2) | 3.330(2) | 3.334(2) | Ca2/Sr2*^a^*-Sb2 | 3.336(2) | 3.339(2) |  |
| Ca2-Sb3 | 3.632(2) | 3.344(2) | 3.341(2) | Ca2/Sr2*^a^*-Sb3 | 3.330(2) | 3.328(2) |  |
| Ca2-Sb4 (× 2) | 3.195(2) | 3.225(2) | 3.227(1) | Ca2/Sr2*^a^*-Sb4 (× 2) | 3.234(2) | 3.234(2) |  |
| Ca2-Sb5 (× 2) | 3.202(2) | 3.227(2) | 3.232(2) | Ca2/Sr2*^a^*-Sb5 (× 2) | 3.238(2) | 3.240(2) |  |
| Zn1-Sb1 | 2.966(2) | 3.025(2) | 3.028(2) | Zn1-Sb1 | 3.032(2) | 3.034(2) |  |
| Zn1-Sb2 (× 2) | 2.728(1) | 2.719(1) | 2.721(1) | Zn1-Sb2 (× 2) | 2.722(2) | 2.721(2) |  |
| Zn1-Sb5 | 2.674(2) | 2.648(2) | 2.648(1) | Zn1-Sb5 | 2.648(2) | 2.648(2) |  |
| Zn2-Sb3 (× 2) | 2.697(2) | 2.715(2) | 2.704(2) | Zn2-Sb3 (× 2) | 2.695(1) | 2.696(2) |  |
| Zn2-Sb4 | 2.836(2) | 2.800(3) | 2.815(3) | Zn2-Sb4 | 2.887(2) | 2.888(2) |  |
| Zn2-Sb5 | 2.864(2) | 2.941(3) | 2.955(3) | Zn2-Sb5 | 2.842(2) | 2.840(2) |  |
| Zn3-Sb3 (× 2) | 2.692(2) | 2.692(1) | 2.694(2) | Zn3/Ag-Sb2 | 2.561(2) | 2.568(2) |  |
| Zn3-Sb4 | 2.893(3) | 2.893(2) | 2.890(2) | Zn3/Ag-Sb4 (× 2) | 2.652(2) | 2.655(2) |  |
| Zn3-Sb5 | 2.924(3) | 2.846(1) | 2.847(2) |  |  |  |  |
| Zn4/Ag-Sb2 | 2.485(2) | 2.536(2) | 2.543(2) |  |  |  |  |
| Zn4/Ag-Sb4 (× 2) | 2.617(2) | 2.642(2) | 2.641(2) |  |  |  |  |
| *^a^*For the Ca/Sr mixed sites, the numbering for Sr follows that of Ca for convenience. | | | | | | | |

| **TABLE S3.** Selected polyhedral volumes (Å^3^) in the Ca_9-_*_x_*Sr*_x_*Zn_4+_*_y_*_-_*_z_*Ag*_z_*Sb_9_ (0 ≤ *x* ≤ 1.41(2); 0.48(2) ≤ *y* ≤ 0.56(2); 0 ≤ *z* ≤ 0.22(3)) system | | | |
| --- | --- | --- | --- |
| Cation site | Volume (Å^3^) | | |
|  | Ca_9_Zn_4.48(2)_Sb_9_ | Ca_8.45(3)_Sr_0.55_Zn_4.28(3)_Ag_0.22_Sb_9_ | Ca_7.59(2)_Sr_1.41_Zn_4.37(2)_Ag_0.19_Sb_9_ |
|  | (Parental Ca_9_Mn_4_Bi_9_-type) | (Intermediate Ca_9_Mn_4_Bi_9_-type) | (Ca_7.6_Sr_1.4_AZn_4.4_Ag_0.2_Sb_9_-type) |
| Ca1 | 46.99 | - | - |
| Ca1/Sr1 | - | 48.19 | 49.79 |
| Ca2 | 45.01 | 45.53 | - |
| Ca2/Sr2 | - | - | 46.25 |

| **TABLE S4.** Structure details of the three hypothetical models of Ca_9_Zn_4.5_Sb_9_, Ca_8_SrZn_4.25_Ag_0.25_Sb_9_, and Ca_7.5_Sr_1.5_Zn_4.25_Ag_0.25_Sb_9_. | | | | | | | |
| --- | --- | --- | --- | --- | --- | --- | --- |
| Chemical formula | | Ca_9_Zn_4.5_Sb_9_ | | Ca_8_SrZn_4.25_Ag_0.25_Sb_9_ | | Ca_7.5_Sr_1.5_Zn_4.25_Ag_0.25_Sb_9_ | |
| Structure type | | Ca_9_Mn_4_Bi_9_-type  (Parental) | | Ca_9_Mn_4_Bi_9_-type  (Intermediate) | | Ca_7.6_Sr_1.4_Zn_4.4_Ag_0.2_Sb_9_-type | |
| Space group | | *Pm* (No. 6) | | | | | |
| Unit cell dimensions (Å) | | *a* = 21.8484 | | *a* = 21.8014 | | *a* = 21.8441 | |
|  | | *b* = 9.0850 | | *b* = 9.109 | | *b* = 9.1210 | |
|  | | *c* = 12.4557 | | *c* = 12.7519 | | *c* = 12.7956 | |
| Volume (Å^3^) | | 2472.37 | | 2518.37 | | 2549.40 | |
| Atom | Wyckoff site | | Atomic coordinates | | | | |
|  |  |  | *x* | | *y* | | *z* |
| Ca_9_Zn_4.5_Sb_9_ | | | | | | | |
| Ca1 | 2*c* | | 0 | | 0.25 | | 0 |
| Ca2 | 2*c* | | 0.5 | | 0.25 | | 0.5 |
| Ca3 | 1*a* | | 0.8622 | | 0 | | 0.1366 |
| Ca4 | 1*b* | | 0.1378 | | 0.5 | | 0.8634 |
| Ca5 | 1*a* | | 0.1378 | | 0 | | 0.8634 |
| Ca6 | 1*b* | | 0.8622 | | 0.5 | | 0.1366 |
| Ca7 | 1*a* | | 0.3622 | | 0 | | 0.3634 |
| Ca8 | 1*b* | | 0.6378 | | 0.5 | | 0.6366 |
| Ca9 | 1*a* | | 0.6378 | | 0 | | 0.6366 |
| Ca10 | 1*b* | | 0.3622 | | 0.5 | | 0.3634 |
| Ca11 | 1*a* | | 0.9087 | | 0 | | 0.4398 |
| Ca12 | 1*b* | | 0.0913 | | 0.5 | | 0.5602 |
| Ca13 | 1*a* | | 0.0913 | | 0 | | 0.5602 |
| Ca14 | 1*b* | | 0.9087 | | 0.5 | | 0.4398 |
| Ca15 | 1*a* | | 0.4087 | | 0 | | 0.0602 |
| Ca16 | 1*b* | | 0.5913 | | 0.5 | | 0.9398 |
| Ca17 | 1*a* | | 0.5913 | | 0 | | 0.9398 |
| Ca18 | 1*b* | | 0.4087 | | 0.5 | | 0.0602 |
| Ca19 | 1*a* | | 0.7391 | | 0 | | 0.3777 |
| Ca20 | 1*b* | | 0.2609 | | 0.5 | | 0.6223 |
| Ca21 | 1*a* | | 0.2609 | | 0 | | 0.6223 |
| Ca22 | 1*b* | | 0.7391 | | 0.5 | | 0.3777 |
| Ca23 | 1*a* | | 0.2391 | | 0 | | 0.1223 |
| Ca24 | 1*b* | | 0.7609 | | 0.5 | | 0.8777 |
| Ca25 | 1*a* | | 0.7609 | | 0 | | 0.8777 |
| Ca26 | 1*b* | | 0.2391 | | 0.5 | | 0.1223 |
| Ca27 | 2*c* | | 0.6026 | | 0.25 | | 0.2109 |
| Ca28 | 2*c* | | 0.3974 | | 0.75 | | 0.7891 |
| Ca29 | 2*c* | | 0.1026 | | 0.25 | | 0.2891 |
| Ca30 | 2*c* | | 0.8974 | | 0.75 | | 0.7109 |
| Zn1 | 2*c* | | 0.9546 | | 0.25 | | 0.2791 |
| Zn2 | 2*c* | | 0.0454 | | 0.75 | | 0.7209 |
| Zn3 | 2*c* | | 0.4546 | | 0.25 | | 0.2209 |
| Zn4 | 2*c* | | 0.5454 | | 0.75 | | 0.7791 |
| Zn5 | 2*c* | | 0.7611 | | 0.25 | | 0.1202 |
| Zn6 | 2*c* | | 0.2389 | | 0.75 | | 0.8798 |
| Zn7 | 2*c* | | 0.2611 | | 0.25 | | 0.3798 |
| Zn8 | 2*c* | | 0.7389 | | 0.75 | | 0.6202 |
| Zn9 | 1*a* | | 0.6029 | | 0 | | 0.3958 |
| Zn10 | 1*b* | | 0.3971 | | 0.5 | | 0.6042 |
| Sb1 | 2*c* | | 0 | | 0.25 | | 0.5 |
| Sb2 | 2*c* | | 0.5 | | 0.25 | | 0 |
| Sb3 | 1*a* | | 0.5035 | | 0 | | 0.3059 |
| Sb4 | 1*b* | | 0.4965 | | 0.5 | | 0.6941 |
| Sb5 | 1*a* | | 0.4965 | | 0 | | 0.6941 |
| Sb6 | 1*b* | | 0.5035 | | 0.5 | | 0.3059 |
| Sb7 | 1*a* | | 0.0035 | | 0 | | 0.1941 |
| Sb8 | 1*b* | | 0.9965 | | 0.5 | | 0.8059 |
| Sb9 | 1*a* | | 0.9965 | | 0 | | 0.8059 |
| Sb10 | 1*b* | | 0.0035 | | 0.5 | | 0.1941 |
| Sb11 | 1*a* | | 0.6948 | | 0 | | 0.1227 |
| Sb12 | 1*b* | | 0.3052 | | 0.5 | | 0.8773 |
| Sb13 | 1*a* | | 0.3052 | | 0 | | 0.8773 |
| Sb14 | 1*b* | | 0.6948 | | 0.5 | | 0.1227 |
| Sb15 | 1*a* | | 0.1948 | | 0 | | 0.3773 |
| Sb16 | 1*b* | | 0.8052 | | 0.5 | | 0.6227 |
| Sb17 | 1*a* | | 0.8052 | | 0 | | 0.6227 |
| Sb18 | 1*b* | | 0.1948 | | 0.5 | | 0.3773 |
| Sb19 | 2*c* | | 0.6476 | | 0.25 | | 0.4598 |
| Sb20 | 2*c* | | 0.3524 | | 0.75 | | 0.5402 |
| Sb21 | 2*c* | | 0.1476 | | 0.25 | | 0.0402 |
| Sb22 | 2*c* | | 0.8524 | | 0.75 | | 0.9598 |
| Sb23 | 2*c* | | 0.8334 | | 0.25 | | 0.3093 |
| Sb24 | 2*c* | | 0.1666 | | 0.75 | | 0.6907 |
| Sb25 | 2*c* | | 0.3334 | | 0.25 | | 0.1907 |
| Sb26 | 2*c* | | 0.6666 | | 0.75 | | 0.8093 |
| Ca_8_SrZn_4.25_Ag_0.25_Sb_9_ | | | | | | | |
| Ca1 | 2*c* | | 0 | | 0.25 | | 0 |
| Ca2 | 2*c* | | 0.5 | | 0.25 | | 0.5 |
| Ca3 | 1*a* | | 0.8457 | | 0 | | 0.133 |
| Ca4 | 1*b* | | 0.1543 | | 0.5 | | 0.867 |
| Ca5 | 1*a* | | 0.1543 | | 0 | | 0.867 |
| Ca6 | 1*b* | | 0.8457 | | 0.5 | | 0.133 |
| Ca7 | 1*a* | | 0.3457 | | 0 | | 0.367 |
| Ca8 | 1*b* | | 0.6543 | | 0.5 | | 0.633 |
| Ca9 | 1*a* | | 0.6543 | | 0 | | 0.633 |
| Ca10 | 1*b* | | 0.3457 | | 0.5 | | 0.367 |
| Ca11 | 1*a* | | 0.9088 | | 0 | | 0.4356 |
| Ca12 | 1*b* | | 0.0912 | | 0.5 | | 0.5644 |
| Ca13 | 1*a* | | 0.0912 | | 0 | | 0.5644 |
| Ca14 | 1*b* | | 0.9088 | | 0.5 | | 0.4356 |
| Ca15 | 1*a* | | 0.4088 | | 0 | | 0.0644 |
| Ca16 | 1*b* | | 0.5912 | | 0.5 | | 0.9356 |
| Ca17 | 1*a* | | 0.5912 | | 0 | | 0.9356 |
| Ca18 | 1*b* | | 0.4088 | | 0.5 | | 0.0644 |
| Ca19 | 1*a* | | 0.7329 | | 0 | | 0.3555 |
| Ca20 | 1*b* | | 0.2671 | | 0.5 | | 0.6445 |
| Ca21 | 1*a* | | 0.2671 | | 0 | | 0.6445 |
| Ca22 | 1*b* | | 0.7329 | | 0.5 | | 0.3555 |
| Ca23 | 1*a* | | 0.2329 | | 0 | | 0.1445 |
| Ca24 | 1*b* | | 0.7671 | | 0.5 | | 0.8555 |
| Ca25 | 1*a* | | 0.7671 | | 0 | | 0.8555 |
| Ca26 | 1*b* | | 0.2329 | | 0.5 | | 0.1445 |
| Ca27 | 2c | | 0.0962 | | 0.25 | | 0.2937 |
| Ca28 | 2c | | 0.9038 | | 0.75 | | 0.7063 |
| Sr1 | 2*c* | | 0.5962 | | 0.25 | | 0.2063 |
| Sr2 | 2*c* | | 0.4038 | | 0.75 | | 0.7937 |
| Zn1 | 2*c* | | 0.9493 | | 0.25 | | 0.2792 |
| Zn2 | 2*c* | | 0.0507 | | 0.75 | | 0.7208 |
| Zn3 | 2*c* | | 0.4493 | | 0.25 | | 0.2208 |
| Zn4 | 2*c* | | 0.5507 | | 0.75 | | 0.7792 |
| Zn5 | 2*c* | | 0.722 | | 0.25 | | 0.0153 |
| Zn6 | 2*c* | | 0.2401 | | 0.75 | | 0.8892 |
| Zn7 | 2*c* | | 0.222 | | 0.25 | | 0.4847 |
| Zn8 | 2*c* | | 0.7401 | | 0.75 | | 0.6108 |
| Zn9 | 1*b* | | 0.4054 | | 0.5 | | 0.5908 |
| Ag | 1*a* | | 0.5946 | | 0 | | 0.4092 |
| Sb1 | 2*c* | | 0 | | 0.25 | | 0.5 |
| Sb2 | 2*c* | | 0.5 | | 0.25 | | 0 |
| Sb3 | 1*a* | | 0.9945 | | 0 | | 0.192 |
| Sb4 | 1*b* | | 0.0055 | | 0.5 | | 0.808 |
| Sb5 | 1*a* | | 0.0055 | | 0 | | 0.808 |
| Sb6 | 1*b* | | 0.9945 | | 0.5 | | 0.192 |
| Sb7 | 1*a* | | 0.4945 | | 0 | | 0.308 |
| Sb8 | 1*b* | | 0.5055 | | 0.5 | | 0.692 |
| Sb9 | 1*a* | | 0.5055 | | 0 | | 0.692 |
| Sb10 | 1*b* | | 0.4945 | | 0.5 | | 0.308 |
| Sb11 | 1*a* | | 0.6926 | | 0 | | 0.1161 |
| Sb12 | 1*b* | | 0.3074 | | 0.5 | | 0.8839 |
| Sb13 | 1*a* | | 0.3074 | | 0 | | 0.8839 |
| Sb14 | 1*b* | | 0.6926 | | 0.5 | | 0.1161 |
| Sb15 | 1*a* | | 0.1926 | | 0 | | 0.3839 |
| Sb16 | 1*b* | | 0.8074 | | 0.5 | | 0.6161 |
| Sb17 | 1*a* | | 0.8074 | | 0 | | 0.6161 |
| Sb18 | 1*b* | | 0.1926 | | 0.5 | | 0.3839 |
| Sb19 | 2*c* | | 0.6502 | | 0.25 | | 0.454 |
| Sb20 | 2*c* | | 0.3498 | | 0.75 | | 0.546 |
| Sb21 | 2*c* | | 0.1502 | | 0.25 | | 0.046 |
| Sb22 | 2*c* | | 0.8498 | | 0.75 | | 0.954 |
| Sb23 | 2*c* | | 0.8291 | | 0.25 | | 0.31 |
| Sb24 | 2*c* | | 0.1709 | | 0.75 | | 0.69 |
| Sb25 | 2*c* | | 0.3291 | | 0.25 | | 0.19 |
| Sb26 | 2*c* | | 0.6709 | | 0.75 | | 0.81 |
| Ca_7.5_Sr_1.5_Zn_4.25_Ag_0.25_Sb_9_ | | | | | | | |
| Ca1 | 2c | | 0 | | 0.25 | | 0 |
| Ca2 | 2*c* | | 0.5 | | 0.25 | | 0.5 |
| Ca3 | 1*a* | | 0.8448 | | 0 | | 0.1329 |
| Ca4 | 1*b* | | 0.1552 | | 0.5 | | 0.8671 |
| Ca5 | 1*a* | | 0.1552 | | 0 | | 0.8671 |
| Ca6 | 1*b* | | 0.8448 | | 0.5 | | 0.1329 |
| Ca7 | 1*a* | | 0.3448 | | 0 | | 0.3671 |
| Ca8 | 1*b* | | 0.6552 | | 0.5 | | 0.6329 |
| Ca9 | 1*a* | | 0.6552 | | 0 | | 0.6329 |
| Ca10 | 1*b* | | 0.3448 | | 0.5 | | 0.3671 |
| Ca11 | 1*a* | | 0.9087 | | 0 | | 0.4357 |
| Ca12 | 1*b* | | 0.0913 | | 0.5 | | 0.5643 |
| Ca13 | 1*a* | | 0.0913 | | 0 | | 0.5643 |
| Ca14 | 1*b* | | 0.9087 | | 0.5 | | 0.4357 |
| Ca15 | 1*a* | | 0.4087 | | 0 | | 0.0643 |
| Ca16 | 1*b* | | 0.5913 | | 0.5 | | 0.9357 |
| Ca17 | 1*a* | | 0.5913 | | 0 | | 0.9357 |
| Ca18 | 1*b* | | 0.4087 | | 0.5 | | 0.0643 |
| Ca19 | 1*a* | | 0.7326 | | 0 | | 0.3544 |
| Ca20 | 1*b* | | 0.2674 | | 0.5 | | 0.6456 |
| Ca21 | 1*a* | | 0.2674 | | 0 | | 0.6456 |
| Ca22 | 1*b* | | 0.7326 | | 0.5 | | 0.3544 |
| Ca23 | 1*a* | | 0.2326 | | 0 | | 0.1456 |
| Ca24 | 1*b* | | 0.7674 | | 0.5 | | 0.8544 |
| Ca25 | 1*a* | | 0.7674 | | 0 | | 0.8544 |
| Ca26 | 1*b* | | 0.2326 | | 0.5 | | 0.1456 |
| Ca27 | 2c | | 0.904 | | 0.75 | | 0.7062 |
| Sr1 | 2*c* | | 0.596 | | 0.25 | | 0.2062 |
| Sr2 | 2*c* | | 0.404 | | 0.75 | | 0.7938 |
| Sr3 | 2*c* | | 0.096 | | 0.25 | | 0.2938 |
| Zn1 | 2*c* | | 0.9488 | | 0.25 | | 0.2797 |
| Zn2 | 2*c* | | 0.0512 | | 0.75 | | 0.7203 |
| Zn3 | 2*c* | | 0.4488 | | 0.25 | | 0.2203 |
| Zn4 | 2*c* | | 0.5512 | | 0.75 | | 0.7797 |
| Zn5 | 2*c* | | 0.722 | | 0.25 | | 0.0149 |
| Zn6 | 2*c* | | 0.278 | | 0.75 | | 0.9851 |
| Zn7 | 2*c* | | 0.222 | | 0.25 | | 0.4851 |
| Zn8 | 2*c* | | 0.778 | | 0.75 | | 0.5149 |
| Zn9 | 1*b* | | 0.4052 | | 0.5 | | 0.5913 |
| Ag | 1*a* | | 0.5948 | | 0 | | 0.4087 |
| Sb1 | 2*c* | | 0 | | 0.25 | | 0.5 |
| Sb2 | 2*c* | | 0.5 | | 0.25 | | 0 |
| Sb3 | 1*a* | | 0.9936 | | 0 | | 0.1923 |
| Sb4 | 1*b* | | 0.0064 | | 0.5 | | 0.8077 |
| Sb5 | 1*a* | | 0.0064 | | 0 | | 0.8077 |
| Sb6 | 1*b* | | 0.9936 | | 0.5 | | 0.1923 |
| Sb7 | 1*a* | | 0.4936 | | 0 | | 0.3077 |
| Sb8 | 1*b* | | 0.5064 | | 0.5 | | 0.6923 |
| Sb9 | 1*a* | | 0.5064 | | 0 | | 0.6923 |
| Sb10 | 1*b* | | 0.4936 | | 0.5 | | 0.3077 |
| Sb11 | 1*a* | | 0.6927 | | 0 | | 0.1155 |
| Sb12 | 1*b* | | 0.3073 | | 0.5 | | 0.8845 |
| Sb13 | 1*a* | | 0.3073 | | 0 | | 0.8845 |
| Sb14 | 1*b* | | 0.6927 | | 0.5 | | 0.1155 |
| Sb15 | 1*a* | | 0.1927 | | 0 | | 0.3845 |
| Sb16 | 1*b* | | 0.8073 | | 0.5 | | 0.6155 |
| Sb17 | 1*a* | | 0.8073 | | 0 | | 0.6155 |
| Sb18 | 1*b* | | 0.1927 | | 0.5 | | 0.3845 |
| Sb19 | 2*c* | | 0.6508 | | 0.25 | | 0.4538 |
| Sb20 | 2*c* | | 0.3492 | | 0.75 | | 0.5462 |
| Sb21 | 2*c* | | 0.1508 | | 0.25 | | 0.0462 |
| Sb22 | 2*c* | | 0.8492 | | 0.75 | | 0.9538 |
| Sb23 | 2*c* | | 0.8289 | | 0.25 | | 0.3105 |
| Sb24 | 2*c* | | 0.1711 | | 0.75 | | 0.6895 |
| Sb25 | 2*c* | | 0.3289 | | 0.25 | | 0.1895 |
| Sb26 | 2*c* | | 0.6711 | | 0.75 | | 0.8105 |

| **TABLE S5.** Comparison of the maximum *ZT* of several derived compounds in the A_9_M_4+_*_x_*Sb_9_ (A = Ca, Sr, Eu, Yb; M = Zn, Cu, Ag) system. | | | | |
| --- | --- | --- | --- | --- |
| Compound | Structure type | Maximum *ZT* | *T* (K) | Reference |
| Ca_8_YbZn_4.4_Cu_0.1_Sb_9_ | Ca_9_Mn_4_Bi_9_-type | 0.81 | 843 K | [6] |
| Ca_8.5_Eu_0.5_Zn_4.4_Cu_0.1_Sb_9_ | Ca_9_Mn_4_Bi_9_-type | 0.60 | 810 K | [7] |
| Ca_8.2_Sr_0.8_Zn_4.5_Sb_9_ | Ca_9_Mn_4_Bi_9_-type | 0.73 | 866 K | [11] |
| Ca_9_Zn_4.45_Ag_0.05_Sb_9_ | Ca_9_Mn_4_Bi_9_-type | 0.60 | 873 K | [13] |
| Ca_9_Zn_4.35_Cu_0.15_Sb_9_ | Ca_9_Mn_4_Bi_9_-type | 0.72 | 873 K | [14] |
| Ca_9_Cd_4.05_Zn_0.45_Sb_9_ | Ca_9_Zn_3.1_In_0.9_Sb_9_-type | 0.70 | 775 K | [34] |
| Ca_7.59(2)_Sr_1.41_Zn_4.37(2)_Ag_0.19_Sb_9_ | Ca_7.6_Sr_1.4_Zn_4.4_Ag_0.2_Sb_9_-type | 0.73 | 873 K | This work |

**FIGURE S1.** Five distorted octahedral cationic sites surrounded by six Sb atoms in the parental phase Ca_9_Zn_4.48(2)_Sb_9_. The volume of each polyhedron is also displayed.

**FIGURE S2.** The Zn3 occupation and the Ca2-Sb3 distance as a function of Sr content in the Ca_9-_*_x_*Sr*_x_*Zn_4+_*_y_*_-_*_z_*Ag*_z_*Sb_9_ (0.55(3) ≤ *x* ≤ 1.43(2); 0.50(3) ≤ *y* ≤ 0.56(2); 0.10(2) ≤ *z* ≤ 0.29(2)) system.

**FIGURE S3.** (a) TDOS, PDOS, and (b) COHP curves of the intermediate phase Ca_8_SrZn_4.25_Ag_0.25_Sb_9_. In the COHP diagram, the “+” region indicates bonding interactions, while the “-” region represents antibonding interactions. Color codes in the DOS curve are as follows: TDOS, black outline; Ca PDOS, gray region; Sr PDOS, pink region; Zn PDOS, cyan region; Ag PDOS, purple region; and Sb PDOS, blue region. *E*_F_ (vertical line) is set to 0 eV, the energy reference with its corresponding VEC of 71.75. The VEC of 71.87 for the corresponding refined compound Ca_8.11(3)_Sr_0.89_Zn_4.33(2)_Ag_0.21_Sb_9_ is also displayed.

**FIGURE S4.** TDOS, PDOS, and COHP of Ca_8_SrZn_4.5_Sb_9_ (a,b) and Ca_9_Zn_4.25_Ag_0.25_Sb_9_ (c,d). In the COHP diagram, the “+” region indicates bonding interactions, while the “-” region represents antibonding interactions. Color codes in the DOS curve are as follows: TDOS, black outline; Ca PDOS, gray region; Sr PDOS, pink region; Zn PDOS, cyan region; Ag PDOS, purple region; and Sb PDOS, blue region. *E*_F_ (vertical line) is set as the energy reference at 0 eV, and the corresponding VEC is also displayed. The VEC of 71.60, corresponding to the refined composition of Ca_9_Zn_4.20(1)_Ag_0.20_Sb_9_, is also displayed.

**FIGURE S5.** Pisarenko plot of *S* versus *n* at room temperature for the four selected compounds in the Ca_9-_*_x_*Sr*_x_*Zn_4+_*_y_*_-_*_z_*Ag*_z_*Sb_9_ (0 ≤ *x* ≤ 1.43(2); 0.50 ≤ *y* ≤ 0.56(2); 0 ≤ *z* ≤ 0.29(2)) system. The solid lines represent the theoretical contours for *m*^∗^ derived from the Mott relationship.

**FIGURE S6.** Temperature-dependent (a) *PF* and (b) *κ*_latt_ of the five title compounds in the Ca_9-_*_x_*Sr*_x_*Zn_4+_*_y_*_-_*_z_*Ag*_z_*Sb_9_ (0.55(3) ≤ *x* ≤ 1.43(2); 0.50(3) ≤ *y* ≤ 0.56(2); 0.10(2) ≤ *z* ≤ 0.29(2)) system measured between 303 and 873 K.

**FIGURE S7.** Temperature-dependent (a) *μ*_w_ and (b) *B* in the Ca_9-_*_x_*Sr*_x_*Zn_4+_*_y_*_-_*_z_*Ag*_z_*Sb_9_ (0 ≤ *x* ≤ 1.43(2); 0.47(2) ≤ *y* ≤ 0.56(2); 0 ≤ *z* ≤ 0.29(2)) system.

**FIGURE S8.** Elemental frequency heatmaps of (a) the generalized Starry dataset and (b) the highly curated ESTM dataset.

**FIGURE S9.** Distribution of crystallographic structural families within (a) the Starry dataset and (b) the ESTM dataset. The curated ESTM dataset exhibits a significantly higher concentration of Zintl phases, including a critical 3.0% representation of the targeted A_9_M_4+_*_x_*Pn_9_-type family.

**FIGURE S10.** Histograms of TE property distributions across the training datasets. Panels (a–c) show the distributions of log_10_*σ*, *S*, and log_10_*κ*_tot_, respectively, for the Starry dataset, while panels (d–f) show the corresponding distributions for the ESTM dataset.

**FIGURE S11.** Temperature-dependent TE property trends across the Starry and ESTM datasets. Panels (a–c) show the temperature-dependent trends of log_10_*σ*, *S*, and log_10_*κ*_tot_, respectively, for the Starry dataset, and panels (d–f) show the corresponding trends for the ESTM dataset. The curated ESTM data successfully captures complex physical phenomena for the A_9_M_4+_*_x_*Pn_9_ family, including degenerate semiconducting behavior, the high-temperature bipolar effect, and Umklapp phonon scattering.

**FIGURE S12.** Latent space Pearson correlation (*r*) heatmaps extracted from the trained models for *σ*, *S*, and *κ*_tot_. The color scale ranges from −0.3 to 1.0 and illustrates the learned chemical similarities between elements.

**FIGURE S13.** 10-fold cross-validation metrics utilizing strict composition-based splitting. Panels (a–c) show the *R*², MAE, and RMSE metrics, respectively, for log_10_*σ*; panels (d–f) show the corresponding metrics for *S*; and panels (g–i) show the corresponding metrics for log_10_*κ*_tot_. The minimal variance in predictive performance across all folds confirms the structural stability of ML models and prevents data leakage for unseen chemical formulas.

**FIGURE S14.** Feature sensitivity analysis for (a) log_10_*σ*, (b) *S*, and (c) log_10_*κ*_tot_, tracking predictive performance *R*² against the number of top XGBoost features.

**FIGURE S15.** Learning curves for the prediction of (a) log_10_*σ*, (b) *S*, and (c) log_10_*κ*_tot_, showing test *R*^2^ and MAE as functions of training-set size over approximately 10–100% of the available training data.

**FIGURE S16.** Quantitative error analysis of the predicted versus experimental TE properties for (a) *σ*, (b) *S*, (c) *κ*_tot_, and (d) *ZT* at 873 K.

**FIGURE S17**. Optical microscopic images of bar/needle-shaped single crystals of (a) Ca_8.11(3)_Sr_0.89_Zn_4.33(2)_Ag_0.21_Sb_9_ and (b) Ca_7.59(2)_Sr_1.41_Zn_4.37(2)_Ag_0.19_Sb_9_ with scale bars. Some remaining Pb metal is observed on the surface of the crystals.

**FIGURE S18**. PXRD patterns of five title compounds in the Ca_9-_*_x_*Sr*_x_*Zn_4+_*_y_*_-_*_z_*Ag*_z_*Sb_9_ (0.55(3) ≤ *x* ≤ 1.43(2); 0.50(3) ≤ *y* ≤ 0.56(2); 0.10(2) ≤ *z* ≤ 0.29(2)) system. For comparison purposes, a simulated PXRD pattern of Ca_7.59(2)_Sr_1.41_Zn_4.37(2)_Ag_0.19_Sb_9_ is also provided as a reference.

**FIGURE S19.** TGA results for Ca_8.45(3)_Sr_0.55_Zn_4.28(3)_Ag_0.22_Sb_9_, Ca_8.11(3)_Sr_0.89_Zn_4.33(2)_Ag_0.21_Sb_9_, and Ca_7.59(2)_Sr_1.41_Zn_4.37(2)_Ag_0.19_Sb_9_ over the temperature range between 300 and 1200 K.

**FIGURE S20.** EDS results of elemental compositions and corresponding mapping images for (a) Ca_7.97(3)_Sr_1.03_Zn_4.42(2)_Ag_0.10_Sb_9_, (b) Ca_7.59(2)_Sr_1.41_Zn_4.37(2)_Ag_0.19_Sb_9_, and (c) Ca_7.57(2)_Sr_1.43_Zn_4.27(2)_Ag_0.29_Sb_9_. The corresponding compositions determined by EDS are approximately Ca_7.82_Sr_1.19_Zn_4.47_Ag_0.08_Sb_8.93_, Ca_7.37_Sr_1.57_Zn_4.36_Ag_0.20_Sb_8.99_, and Ca_7.42_Sr_1.57_Zn_4.30_Ag_0.30_Sb_8.90_, respectively.

**References**

S1. Bruker, APEX3, Bruker AXS Inc., Madison, WI, USA 2016.

S2. Bruker, *SAINT*, Bruker AXS Inc., Madison, WI, USA 2002.

S3. G. M. Sheldrick, *SADABS*, University of Göttingen, Göttingen, Germany 2003.

S4. L. M. Gelato and E. Parthé, “STRUCTURE TIDY – a Computer Program to Standardize Crystal Structure Data,” *Journal of Applied Crystallography* **20** (1987): 139–143, <https://doi.org/10.1107/S0021889887086965>

S5. D. D. Koelling and B. N. Harmon, “A Technique for Relativistic Spin-Polarised Calculations,” *Journal of Physics C: Solid State Physics* **10** no.10 (1977): 3107, <https://doi.org/10.1088/0022-3719/10/16/019>

S6. O. K. Andersen, O. Jepsen, and D. Glötzel, 1985, “Canonical Description of the Band Structures of Metals,” *Highlights of Condensed Matter Theory*, Elsevier Science, North Holland, NY (1985).

S7. O. Jepsen and O. K. Andersen, “Calculated Electronic Structure of the Sandwich d^1^ Metals LaI_2_ and CeI_2_: Application of New LMTO Techniques,” *Zeitschrift für Physik B Condensed Matter* **97** (1995): 35–47, <https://doi.org/10.1007/BF01317585>

S8. K. A. Borup, J. de Boor, H. Wang, et al., “Measuring Thermoelectric Transport Properties of Materials,” *Energy & Environmental Science* **8**, no. 2 (2015): 423–435, <https://doi.org/10.1039/C4EE01320D>

S9. M. T. Agne, P. W. Voorhees and G. J. Snyder, “Phase Transformation Contributions to Heat Capacity and Impact on Thermal Diffusivity, Thermal Conductivity, and Thermoelectric Performance,” *Advanced Materials* **31,** no.35,(2019): 1902980, <https://doi.org/10.1002/adma.201902980>

S10. K. C. Lukas, W. S. Liu, G. Joshi, et al., “Experimental Determination of the Lorenz Number in Cu_0.01_Bi_2_Te_2.7_Se_0.3_ and Bi_0.88_Sb_0.12_,” *Physical Review B* **85** (2012): 205410, <https://doi.org/10.1103/PhysRevB.85.205410>

S11. L. J. van der Pauw, “A Method of Measuring Specific Resistivity and Hall Effect of Discs of Arbitrary Shape,” *Philips Research Reports* **13** (1958): 1–9.

S12. L. J. van der Pauw, “A Method of Measuring the Resistivity and Hall Coefficient on Lamellae of Arbitrary Shape,” *Philips Technical Review* **20** (1958): 220–224.

S13. R. Kohavi, “A Study of Cross-Validation and Bootstrap for Accuracy Estimation and Model Selection,” *Proceedings of the Fourteenth International Joint Conference on Artificial Intelligence (IJCAI)* (1995): 1137–1145.

S14. S. P. Ong, W. D. Richards, A. Jain, et al., “Python Materials Genomics (pymatgen): A Robust, Open-Source Python Library for Materials Analysis,” *Computational Materials Science* **68** (2013): 314–319, <https://doi.org/10.1016/j.commatsci.2012.10.028>

S15. F. Ottomano, G. De Felice, V. V. Gusev and T. D. Sparks, “Not as Simple as We Thought: A Rigorous Examination of Data Aggregation in Materials Informatics,” *Digital Discovery* **3,** no.2 (2024): 337–346, <https://doi.org/10.1039/D3DD00207A>

S16. S. G. Baird, M. Liu and T. D. Sparks, “High-Dimensional Bayesian Optimization of 23 Hyperparameters over 100 Iterations for an Attention-Based Network to Predict Materials Property: A Case Study on CrabNet Using Ax Platform and SAASBO,” *Computational Materials Science* **211** (2022): 111505, <https://doi.org/10.1016/j.commatsci.2022.111505>

S17. A. Paszke, S. Gross, F. Massa, A. Lerer, J. Bradbury, et al., “PyTorch: An Imperative Style, High-Performance Deep Learning Library,” *Advances in Neural Information Processing Systems* **32** (2019).

S18. T. Akiba, S. Sano, T. Yanase, T. Ohta, M. Koyama, “Optuna: A Next-Generation Hyperparameter Optimization Framework,” *Proceedings of the 25th ACM SIGKDD International Conference on Knowledge Discovery & Data Mining* (2019): 2623–2631, <https://doi.org/10.1145/3292500.3330701>

S19. C. R. Harris, K. J. Millman, S. J. van der Walt, et al., “Array Programming with NumPy,” *Nature* **585** (2020): 357–362, [https://doi.org/10.1038/s41586-020-2649-2](https://doi.org/10.1038/s41586-020-2649-2?utm_source=chatgpt.com)

S20. S. Chetlur, C. Woolley, P. Vandermersch, et al., “cuDNN: Efficient Primitives for Deep Learning,” *arXiv* (2014): 1410.0759, <https://doi.org/10.48550/arXiv.1410.0759>

S21. N. Srivastava, G. Hinton, A. Krizhevsky, I. Sutskever, R. Salakhutdinov, “Dropout: A Simple Way to Prevent Neural Networks from Overfitting,” *Journal of Machine Learning Research* **15** (2014): 1929–1958
